# Targeting the plasticity of intestinal neutrophils: bidirectional regulation strategies by natural products

**DOI:** 10.3389/fimmu.2025.1754107

**Published:** 2026-01-12

**Authors:** Ruotong Kang, Anqi Sun, Jiayin Yang, Linyuan Chang, Wenguang Sun, Fushun Kou, Yuan Cheng

**Affiliations:** 1 School of Integrative Medicine, Nanjing University of Chinese Medicine, Nanjing, China; 2 Jiangsu Collaborative Innovation Center of Traditional Chinese Medicine Prevention and Treatment of Tumor, the First Clinical Medical College, Nanjing University of Chinese Medicine, Nanjing, China

**Keywords:** cell interactions, intestinal immune diseases, intestinal neutrophils, natural products, novel therapeutic techniques

## Abstract

Historically, neutrophils have been regarded primarily as pro-inflammatory cells, yet recent advancements have revealed their phenotypic heterogeneity and functional plasticity with versatile immunophenotypes. Distinct subpopulations of neutrophils exhibit a functional duality, not only initiating and amplifying inflammation, but also actively promoting tissue restoration in diseases, such as inflammatory bowel disease (IBD), colorectal cancer (CRC) and intestinal infections. They contribute to the formation of a dynamic immune microenvironment in concert with the intestinal microbiota, epithelial cells, and other immune cell types. Current first-line therapies for enteric diseases often lack precision in modulating neutrophil functions. In contrast, natural products including alkaloids, polysaccharides, polyphenols, quinones, and glycosides, as well as microbiota-derived metabolites, exhibit distinct advantages for ability to achieve multi-targeted and bidirectional immunomodulation. These compounds target neutrophil activation, migration, neutrophil extracellular trap formation, cytokine release, oxidative stress, and energy metabolism etc. In this review, we systematically examine the heterogeneity and functional diversity of intestinal neutrophils, highlighting their interaction mechanisms with the surrounding microenvironment. Potential of natural products to modulate neutrophil functions via multi-target strategies has not been fully explored. Moreover, the review discusses novel precision therapeutic approaches based on neutrophil nanotechnology and engineered cell drug delivery. These cutting-edge technologies aim to enhance natural products delivery to inflammatory sites, provide controllable regulation of neutrophil function, and facilitate the degradation of pathological structures. Collectively, the study presents new research directions and theoretical frameworks for intervention of natural products in neutrophils of intestinal immune-related disorders, notably IBD and even CRC.

## 1 Introduction

The intestinal immune system is critical in maintaining systemic homeostasis and protecting against pathogenic invasion. Its dysfunction could lead to the development of various complex immune-mediated intestinal disorders. Multiple immune cell types, including lymphocytes, macrophages, dendritic cells, and neutrophils, coordinate interplay to sustain intestinal immune homeostasis (1). Neutrophils, as the primary effector cells of innate immunity, are a key effector response to tissue infection and inflammation. Recent studies have highlighted their remarkable plasticity and functional heterogeneity within the gut, expanding our understanding of their role beyond innate defense (2). Intestinal neutrophils refer to neutrophils that are recruited from the circulation into the intestinal mucosa in response to homeostatic or inflammatory signals. Shaped by the local microenvironment, particularly the microbiota, they adopt specific functional states. Upon intestinal homeostasis disruption, chemokines and cytokines guide neutrophils into intestinal tissue (3), where they undergo transcriptomic and phenotypic reprogramming into distinct functional subsets (4, 5). Although whether there exist tissue-resident neutrophil populations like macrophages remains controversial, evidence indicates that circulating neutrophils can adapt and persist locally. For instance, subsets such as CXCR4^+^ neutrophils exhibit transcriptional profiles skewed toward non-immune regulatory roles (6, 7). Further support comes from the phenomenon of “reverse transendothelial migration, “ wherein tissue-reprogrammed neutrophils re-enter circulation (8).

Research has identified distinct functional subsets of intestinal neutrophils. These neutrophil subsets engage in complex interactions with the gut microbiota, epithelial cells, and other immune cells through processes such as migration, phagocytosis, neutrophil extracellular trap formation (NETs) formation, and cytokine production (9, 10). The dynamic equilibrium of neutrophil subset quantity, function, and spatial distribution is a critical determinant of intestinal immune homeostasis. Disruption of this balance can contribute to the pathogenesis and exacerbation of several intestinal immune disorders, particularly inflammatory bowel disease (IBD) and colorectal cancer (CRC) (9).

Current therapeutic approaches for these conditions primarily involve anti-inflammatory agents (such as aminosalicylates), corticosteroids, and biologics (e.g., anti-TNF-α monoclonal antibodies and interleukin inhibitors) (11). The medicinal value of aspirin, a conventional anti-inflammatory medication, in these scenarios has recently been investigated. Current statistical evidence implies that low-dose aspirin (160mg/d) as a supplementary treatment could diminish the likelihood of CRC returning through the PI3K pathway, although its exact modes of action are not yet fully elucidated (12). These treatments aim to control inflammation, alleviate symptoms, and protect the intestinal mucosa by broadly or selectively inhibiting immune activation (13). Still, most of these therapies have notable limitations and side-effects. Specifically, long-term corticosteroid use is associated with severe metabolic and infective complications, whereas biologics are hampered by high cost, variable efficacy, and potential safety risks (14, 15). Furthermore, these therapies tend to modulate neutrophil function in a non-selective manner, which makes it challenging to effectively suppress excessive NETs formation and oxidative burst of pathogenic neutrophils while preserving or enhancing their pathogen-clearance and tissue-repair abilities (16). As a critical element of the immune defense mechanism, NETs have become a major focus for developing direct pharmacological interventions. Notably, PAD4 inhibitors, including GSK484 and Cl-amidine, can block NETs formation by inhibiting histone citrullination (17, 18). Similarly, DNase I degraded the DNA scaffold of existing NETs, and alleviated NETs-mediated immunothrombosis and tissue injury (19). Furthermore, studies indicated that PAD4 inhibitors and DNase I synergistically reduce hepatic metastasis and peritoneal dissemination in CRC models (20). Although the neutrophil elastase inhibitor sivelestat is primarily used for acute lung injury (21), it may theoretically influence NETs formation and require experimental validation in intestinal diseases. Nevertheless, such drugs are often limited by single targets, short half-lives, potential interference with normal anti-infective immunity, and unverified long-term safety (22, 23), precluding their adoption as standard therapies.

Natural products and their derived compounds, encompassing alkaloids, quinones, polysaccharides, polyphenols, glycosides, terpenoids, microbiota-derived metabolites, which offer a promising alternative therapeutic strategy for the bidirectional regulation of neutrophils due to their multi-target mechanisms, low toxicity, and favorable safety profiles (24). These compounds have garnered attention for their potential in bidirectional immune modulation. Numerous studies have demonstrated that natural products can precisely intervene in neutrophil-mediated inflammatory processes and tissue damage (25). Notably, the effects of natural products are not confined to single targets. They also indirectly modulate neutrophil function by influencing epithelial barrier, T-cell differentiation, and the gut microbiota, thereby demonstrating a multi-layered, network-based immunomodulatory potential (26). In addition, emerging strategies point to targeting neutrophils to enhance the therapeutic precision and efficacy of natural products in intestinal immune diseases (27).

Taken together, the review aims to systematically elucidate the heterogeneity and functional diversity of intestinal neutrophils, along with their complex interactions within the intestinal microenvironment. It particularly focuses on how natural products precisely and multi-targetedly modulate neutrophil functions. The objective is to provide a robust theoretical framework and forward-looking insights to guide the development of novel therapeutic strategies that leverage natural products for the precise and bidirectional regulation of neutrophils.

## 2 Heterogeneity and functional diversity of intestinal neutrophils

### 2.1 Classification, phenotypic characteristics, and function of intestinal neutrophil subpopulations

Different neutrophil subtypes possess specific surface markers, which serve as key indicators for distinguishing between subtypes. The classification of intestinal neutrophil subpopulations (N1, N2, N3), which is based on their function and activation state, should not be conflated with that of tumor-associated neutrophils (TANs, also termed N1 and N2). In fact, their functional roles are often opposing (**Table 1**).

**Table 1:** Classification, phenotypic characteristics and functions of different subtypes of neutrophils in the intestinal tract.

Basis of classification	Subtype	Phenotypic characteristics	Function	Implications in disease	References
Function & Activation State	N1	High expression of CD66b, CD11b, CD16; highly activated, pro-inflammatory	Phagocytosis, ROS production, NETosis, bactericidal, tissue damage	Acute infection, active IBD	(28)
	N2	High expression of CXCR4, CD63; anti-inflammatory and pro-repair	Anti-inflammatory, immunomodulatory and pro-reparative effects	IBD remission, tissue repair	
	N3	Similar to bone marrow mature neutrophils; immunomodulatory	Potential role in immune homeostasis (function under investigation)	Present in IBD	(9)
Density-based subgroups	LDNs	Low density; high expression of CD11b, CD16 or immature phenotypes	Immunosuppressive or pro-inflammatory	Autoimmunity, sepsis, cancer, and other chronic inflammatory conditions	(29, 30)
	NDNs	Conventional neutrophils; altered in disease	Chemotaxis, phagocytosis, antibacterial	Health and disease	(31)
	HDNs	Mature; similar to NDNs, but resting state	Classical neutrophil functions (chemotaxis, phagocytosis, bactericidal activity)	Minimal in health, resting state	(32)
Tumor-Associated Neutrophils (TANs)	N1 (Anti-tumor)	IFN-β-driven; high expression of Fas, TRAIL, ICAM-1, low Arg1 activity	Phagocytosis, cytotoxicity, T cell recruitment, production of TNF-α/ROS, antibody-dependent cellular cytotoxicity (ADCC), immune activation	Anti-tumor in inflammation and cancer	(33, 34)
	N2 (Pro-tumor)	TGF-β-driven; high expression of CXCR4, VEGF, MMP9, high Arg1 activity	T cell suppression, promotion of angiogenesis and metastasis, NETosis	Tumor progression, immunosuppression	
Surface Marker-Based	CD177+	Releases ROS/AMPs/NETs; produces IL-22/TGF-β	Antibacterial, immunomodulation, microbiota interaction	Dysregulation exacerbates inflammation	(35, 36)
	OLFM4+	OLFM4 protein expression	Modulates NETs, antibacterial (controversial)	Biomarker in UC, epithelial injury	(37)
Maturity	Segmented Neutrophils (PMNs)	Fully differentiated, segmented nucleus (2–5 lobes)	Chemotaxis, phagocytosis, antibacterial	Increased in infection/inflammation	(28)
	Band Neutrophils	Immature, basic function	Functional but less efficient chemotaxis and phagocytosis compared to segmented neutrophils	Left shift in acute infection/IBD	(38)
	Metamyelocytes	Immature, not fully functional	Maturation in bone marrow	Severe left shift in infection/leukemia	(39)

### 2.2 Dual mechanisms of different neutrophil subtypes in various diseases

Neutrophils play a complex dual role in intestinal diseases and are regulated by specific subtypes. In IBD, neutrophils are recruited to the mucosa in response to inflammatory signals, where they release reactive oxygen species (ROS), proteolytic enzymes, pro-inflammatory cytokines, and NETs. These actions contribute to epithelial barrier breakdown, crypt abscess formation, and amplifying inflammation (3, 40, 41). However, excessive NETs deposition is associated with an increased risk of thrombosis in active IBD, particularly UC, although it may also provide some protective hemostatic functions (42). Furthermore, neutrophils promote the progression of UC colitis and even transform into colitis-associated carcinomas (CAC) through the activation of the JAK/STAT pathway (43, 44). Conversely, specific subsets such as CD177^+^ neutrophils enhance bactericidal activity and produce tissue-healing factors, thereby supporting mucosal repair (35, 36, 45). Similarly, CXCR4^high^ neutrophils contribute to tissue remodeling via MMP9 secretion (46, 47). In addition, neutrophils produce pro-resolving lipid mediators, vascular endothelial growth factor (VEGF), and MCPIP1, which collectively represent another mechanism to limit excessive inflammation and maintain tissue homeostasis (48).

Within the intestinal tumor microenvironment, the function of neutrophils is influenced by local cytokines and displays context-dependence. In response to stimulation of different cytokines, such as TGF-β, IFN-γ, and IFN-β, neutrophils have the potential to polarize toward an antitumorigenic phenotype (N1) or toward a protumorigenic phenotype (N2) (34, 49–51). N1 neutrophils exert anti-tumor effects through phagocytosis, ROS-mediated cytotoxicity, and recruitment of T cells. N2 neutrophils promote tumor progression by suppressing T cell activity, facilitating angiogenesis via VEGF signaling, and promoting metastasis through MMP9 and NETosis (52, 53). This pro-tumorigenic NETosis can be potently induced by specific members of the dysbiotic intestinal microbiota. A key example is *Fusobacterium nucleatum*, a pathobiont frequently enriched in CRC. It has been demonstrated to trigger NETs formation, which subsequently enhances tumor cell proliferation and invasiveness, thereby facilitating CRC progression (54). This exemplifies a direct mechanistic link between a dysbiotic bacterium, neutrophil activation, and tumor progression.

Moreover, the functional duality of neutrophils extends to other intestinal disorders. In infectious enteritis, neutrophils provide essential defense against pathogens like Salmonella through phagocytosis, ROS production, and NETs formation (55–57). However, excessive activation can exacerbate tissue damage, as seen in Clostridium difficile infection (58). In irritable bowel syndrome, neutrophils contribute to barrier disruption and chronicity, whereas in intestinal obstruction and necrotizing enterocolitis, their early bactericidal activity may be offset by ROS and protease-mediated injury (59). In diverticulitis, rapid neutrophil recruitment localizes infection and prevents peritonitis, albeit at the cost of local tissue destruction (60–62).

## 3 Mechanisms of interaction between neutrophils and the microbiome and other cells

Neutrophils are of crucial significance for maintaining intestinal homeostasis and could influence the intestine through three primary interaction mechanisms, including neutrophil-gut microbiota interactions, neutrophil-epithelial cell interactions, and neutrophil-immune cell interactions.

### 3.1 Neutrophils-gut microbiota

**Figure 1:**
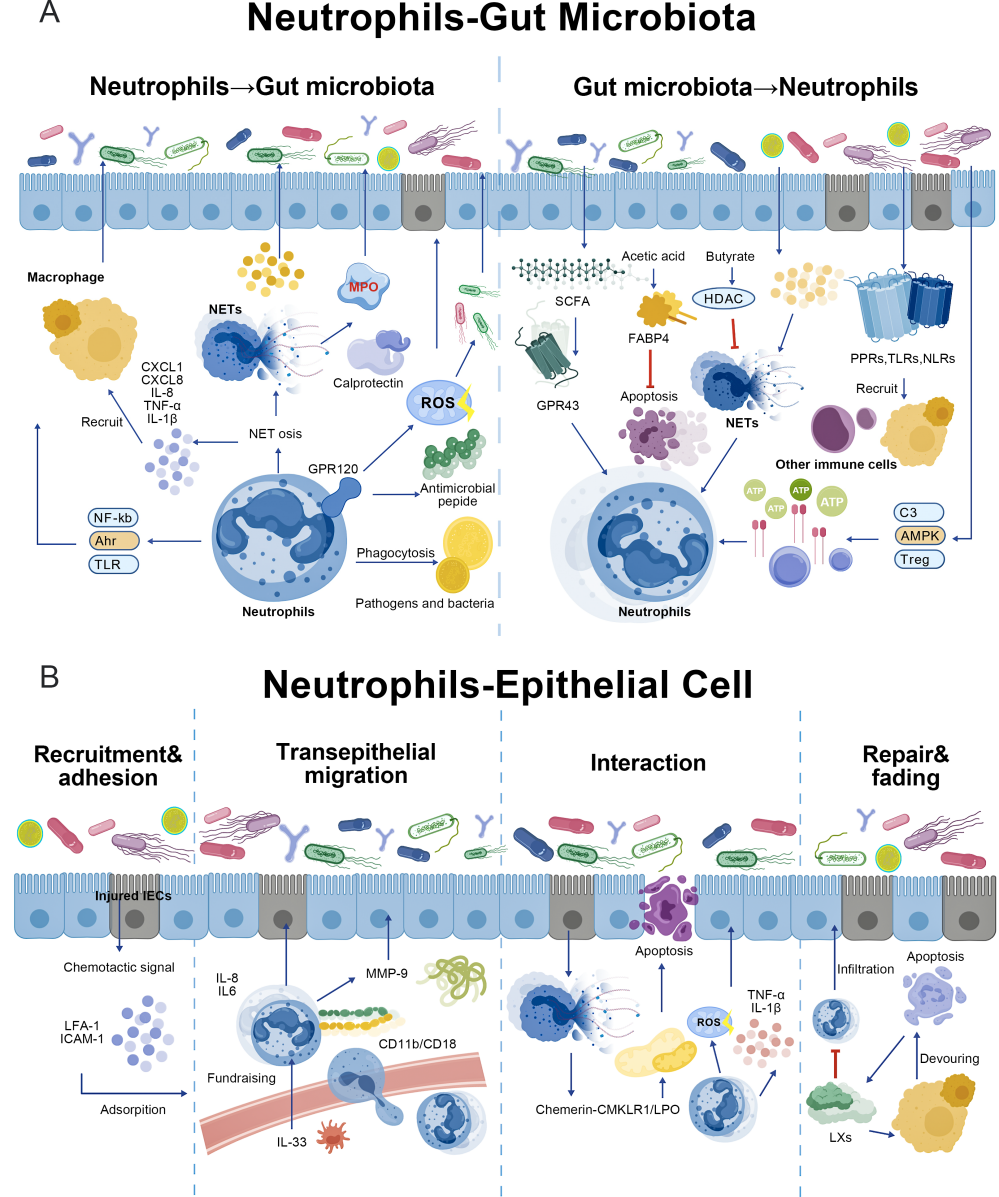
The interaction mechanism between neutrophils and gut microbiota, epithelial cells. **(A)** Neutrophils act on the microbiota through phagocytosis, the release of ROS, antimicrobial peptides (AMPs), and NETs, or through signaling pathways, inflammatory pathways, and immune regulation. Beneficial metabolites in the intestinal microbiota regulate the chemotaxis, apoptosis and NETosis of neutrophils, while pathogen-related molecules strongly activate neutrophils or indirectly regulate them through signaling pathways. **(B)** Neutrophils and epithelial cells interact through inflammatory pathways, signaling pathways, immune regulation and apoptosis in the four stages of recruitment and adhesion, transepithelial migration, epithelial-site interaction, and repair and regression.

Neutrophils directly or indirectly impact the local gut microbiota via multiple pathways (**Figure 1**). Their phagocytic activity eliminates pathogens and dysbiotic commensals (63). Activated neutrophils release antimicrobial peptides and ROS via receptors such as GPR120 to eliminate bacteria (38, 57, 64). In addition, NETs can directly capture and kill microorganisms during NETosis (65, 66). Furthermore, NETosis itself can be mediated by the inflammasome, leading to the release of chemokines and cytokines that indirectly affect the microbiota (58, 67). Neutrophils can also activate the NF-κB signaling pathway through TLR receptors and help counteract dysbiosis through the upregulation of AHR-mediated α-defensin 1 (68, 69). Oxidative stress from neutrophils may suppress anaerobes and promote aerotolerant pathogens, worsening dysbiosis (66, 70). This dysbiosis-driven process can exert influence on tumor microenvironments beyond the gut. For instance, a recent single-cell analysis of CRC peritoneal metastasis revealed that intestinal microbiota dysbiosis promotes the recruitment of inflammatory, pro-tumorigenic neutrophils, which in turn drive mesenchymal transition of malignant and mesothelial cells, thereby facilitating metastatic spread (71). This exemplifies a direct link between dysbiosis, neutrophil recruitment, and tumor progression. Conversely, the intestine microbiota precisely regulates neutrophil function through metabolic products and molecular signaling. Specific microbial metabolites influence neutrophil metabolism (72). (Refer to Section 3.8 for details.) The microbiota also employs multiple indirect pathways to fine-tune neutrophil recruitment, activation, and function. These, including the C3 complement pathway (73), AMPK signaling (74), the CXCL3-PD-L1 axis (75), inflammasome activity (76), and Treg cell differentiation (77), form a complex network of immune microenvironment interactions.

### 3.2 Neutrophil-epithelial cell

The interaction between neutrophils and intestinal epithelial cells (IECs) can be divided into four key processes. Recruitment and adhesion: Under inflammatory stimuli, IECs release chemokines (78, 79), promoting neutrophil adhesion to and retention within the epithelium via adhesion molecules such as LFA-1/ICAM-1 (80, 81). Transmigration: Neutrophils migrate from the lamina propria to the epithelium guided by IL-8, IL-6, IL-33 (82–84), and other factors, utilizing proteases such as CD11b/CD18 and MMP-9 to cleave tight junction proteins and form migration channels (78, 85). Interaction: Neutrophils interact with IECs at injury sites through NETs release, ROS, degranulation, and pro-inflammatory factors (86, 87). At the same time, IECs modulate neutrophil antimicrobial responses and infiltration levels via the chemerin-CMKLR1/LPO axis (88). Resolution of Inflammation: During resolution, neutrophils undergo apoptosis or release pro-resolving mediators, and thus facilitate tissue repair (89–91). It is noteworthy that excessive neutrophil activity or dysregulated defensins can exacerbate mucosal damage (92).

### 3.3 Neutrophils-immune cells

#### 3.3.1 Neutrophils-myeloid immune cells

**Figure 2:**
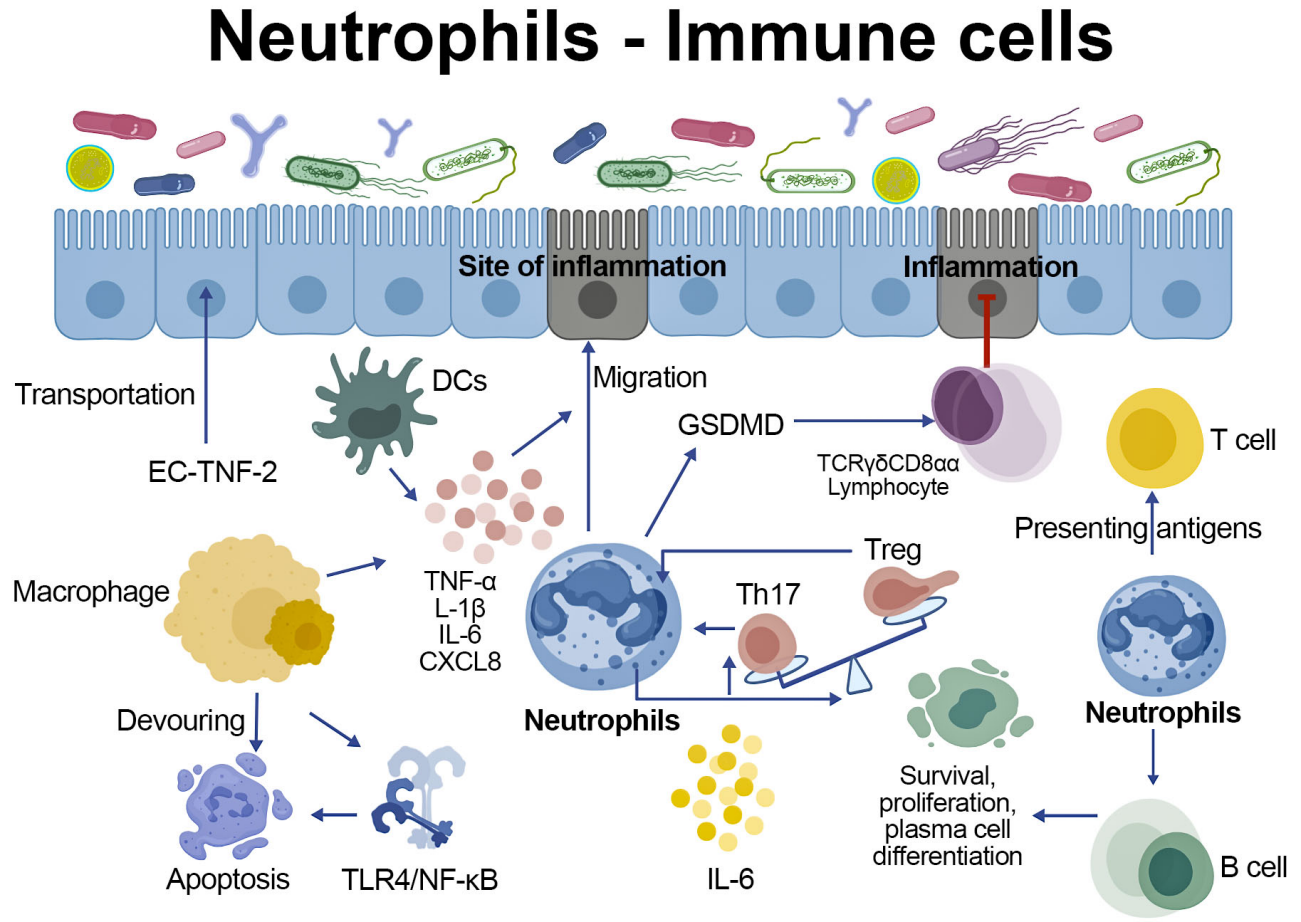
The interaction relationships between neutrophils and myeloid, lymphoid immune cells.Neutrophils act on macrophages, DCs, T cells and B cells through immune regulation, pyroptosis, apoptosis and inflammatory pathways. Myeloid immune cells activate neutrophils in the early stage and phagocytize apoptotic neutrophils in the later stage of inflammation.

The recruitment of neutrophils is regulated by myeloid immune cells such as monocytes, macrophages, and dendritic cells (DCs). In the pre-inflammatory stage, macrophages and DCs produce factors such as CXCL8 and TNF-α that stimulate neutrophil recruitment (93) (**Figure 2**). Neutrophils amplify recruitment signals to attract macrophages and DCs, forming a feedback loop. Macrophages also influence neutrophil trafficking in the inflamed mucosa by modulating the endothelial cell (EC) TNFR2 axis (81). When neutrophils and macrophages reach the site of inflammation, they cooperate in phagocytosis and antigen presentation. During the resolution phase of inflammation, macrophages not only drive necroptosis of neutrophils by inhibiting the TLR4/NF-κB pathway (94), but also efficiently clear apoptotic neutrophils through phagocytosis (95). Failed clearance of apoptotic neutrophils leads to secondary necrosis and persistent inflammation. In addition, the metabolic reprogramming cooperation between neutrophils and macrophages can also regulate inflammatory responses (96).

#### 3.3.2 Neutrophil-lymphoid immune cells

Neutrophils suppress intestinal inflammation by regulating TCRγδCD8αα-expressing intestinal lymphocyte activation through GSDMD-mediated pyroptosis (35). IL-6 drives Th17 differentiation, and Th17-derived chemokines recruit neutrophils in adaptive immunity (97, 98). In the gut, neutrophils play a crucial role in maintaining the balance between Th17 cells and Treg cells. Meanwhile, lymphocytes also exert feedback regulation on neutrophils (77). Additionally, neutrophils can directly present antigens to T cells under certain conditions (99, 100). In CRC, the interaction between CD15^+^ neutrophils and CD8^+^ T cells is linked to tumor progression (101). Neutrophils further support B cell survival, proliferation, and plasma cell differentiation, enhancing IgA production in the mucosa (102).

## 4 Regulatory effects of natural products targeting intestinal neutrophils

### 4.1 Alkaloids

**Figure 3:**
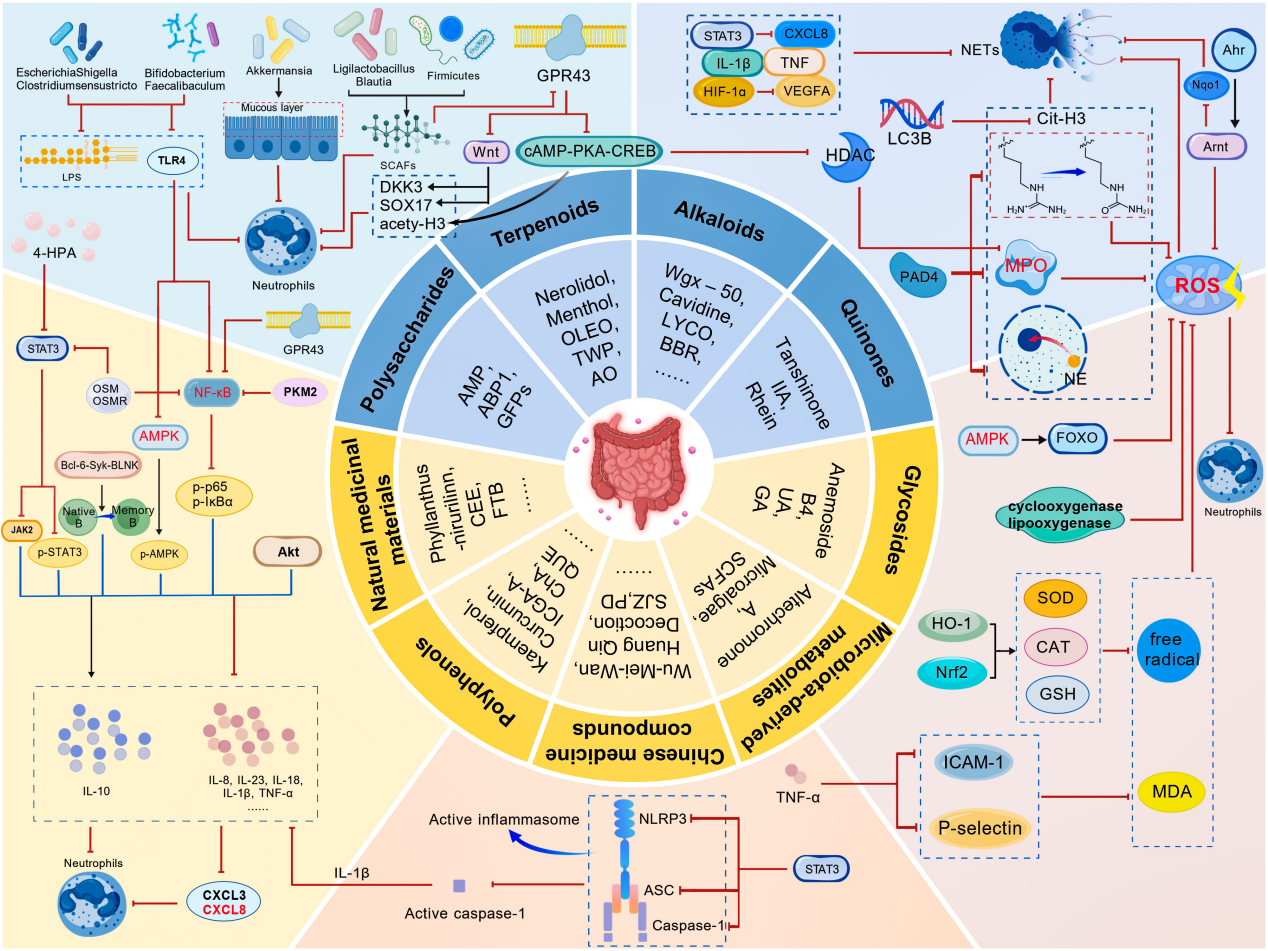
A schematic overview of natural products regulating intestinal neutrophils is described in detail. These natural products are categorised into nine classes: alkaloids, quinones, polysaccharides, polyphenols, glycosides, terpenoids, microbiota-derived metabolites, natural medicinal materials, and traditional Chinese medicine compounds.The section outside the circle illustrates five common pathways through which natural products intervene in intestinal neutrophils: oxidative stress, pyroptosis, microbiota effects, downstream inflammatory factors influenced by the NF-κB pathway and NETs.

Alkaloids are a class of nitrogen-containing organic compounds that occur widely in nature, primarily in plants. These compounds exhibit a range of significant biological activities, including antibacterial, antioxidant, anti-inflammatory, and anti-tumor effects (**Figure 3**, **Table 2**).

**Table 2:** Overview of natural products acting in the gut neutrophil.

Natural products			Disease/research model	Regulation of neutrophil functions	Other regulatory functions	Regulation of disease phenotype	References
Alkaloids	Berberine		CPT11‐induced mice model of intestinal mucositis; Dextran sulphate sodium (DSS)-induced mice model of IBD	The interaction of LINC00668 with NE↓, the nuclear translocation of NE↓; neutrophil activation and infiltration↓	TJs (ZO-1, Claudin-7, Occludin)↑; COX-2↓, iNOS↓, IL-8↓; GUS activity↓, SN38↓	Repair the intestinal barrier function; Anti-inflammatory effects; Regulate the “epithelial - neutrophil” interaction	(86, 103)
	Berbamine		DSS-induced mice model of UC	PAD4↓, Cit-H3↓, MPO↓, NE↓, NETs↓; neutrophil activation and infiltration↓	IL-1β, TNF-α↓	Anti-inflammatory effects	(104)
	Lycopodium		Acetic acid (AA)-induced model of IBD in rats	MPO↓, calprotectin↓, neutrophil activation, recruitment and infiltration↓	IL-1β↓, IL-23↓, NF-κB↓; GSH↑, SOD↑, Catalase (CAT)↑, MDA↓	Anti-inflammatory effects; Reduce oxidative stress	(105)
	Tetramethylpyrazine		Sodium taurocholate-induced ANP model in rats	MPO↓, neutrophil infiltration↓	Ca^2+^↓, ROS↓	Reduce oxidative stress	(106)
	Lemairamin (Wgx-50)		DSS immersion to establish a zebrafish colitis model	Neutrophil recruitment↓	Akt↓, pro-inflammatory cytokines (IL-1β, IL-6, CXCL8a, TNF-α)↓	Anti-inflammatory effects	(107)
	Cavidine		AA-induced model of UC in rats	Neutrophil activation and migration↓	GSH↑, SOD↑, MDA↓, ROS↓; NF-κB↓, TNF-α↓, IL-6↓	Reduce oxidative stress; Anti-inflammatory effects	(108)
Quinones	TanshinoneIIA		Azoxymethane (AOM)/DSS-induced murine CRC model	MPO↓, neutrophil activation↓; migration and infiltration↓	Ly6G↓, ROS↓; intestinal permeability↓; NF-κB↓, p-p65 and p-IκBα↓, pro-inflammatory cytokines (IL-1β, IL-6, IL-10, IL-17A, IFN-γ and TNF-α)↓	Reduce oxidative stress; Repair the intestinal barrier function; Anti-inflammatory effects	(109)
	Rhein		Tail-cutting-induced zebrafish inflammatory models	Neutrophil migration, activation and infiltration↓	NF-κB↓, p-p65↓, proinflammatory cytokines (IL-6, IL-1β, TNF-α)↓, iNOS↓, COX-2↓, NO↓; NLRP3↓, cleaved IL-1β↓	Anti-inflammatory effects; Anti-pyroptotic effects	(110)
Polysaccharides	A. macrocephalae polysaccharides (AMP)		DSS-induced mice model of acute colitis	Neutrophil infiltration, migration and aggregation↓	TJs (ZO-1, Claudin-7, Occludin)↑, MUC-2↑; TNF-α↓, IL-1β↓, IL-6↓; beneficial bacteria (Bifidobacterium, Faecalibaculum)↑, harmful bacteria (EscherichiaShigella, Clostridiumsensustricto1)↓	Repair the intestinal barrier function; Anti-inflammatory effects; Regulate the “microbiota-neutrophil” interaction	(111)
	Agaricus blazei Murill		Temporary superior mesenteric artery occlusion-induced mouse intestinal I/R model	Neutrophil recruitment↓	TJs (ZO-1, Occludin)↑; NLRP3↓, TNF-α↓, IL-1β↓; SCFA producing bacteria (Ligilactobacillus, Blautia)↑, butyrate↑; ROS↓	Repair the intestinal barrier function; Anti-pyroptotic effects; Regulate the “microbiota-neutrophil” interaction; Reduce oxidative stress	(112)
	Grifola frondosa polysaccharides		An oxazolone-induced mice model of UC	Neutrophil infiltration and migration↓	Anti-inflammatory cytokine (IL-10)↑, pro-inflammatory cytokines (IL-6 and TNF-α)↓	Anti-inflammatory effects	(113)
Polyphenols	Forsythiaside A		DSS-induced mice model of UC	PAD4↓, Cit-H3↓, MPO↓, NETs↓; neutrophil infiltration↓	IL-1β↓, TNF-α↓; SCFAs↑	Anti-inflammatory effects; Regulate gut microbiota	(114)
	Isochlorogenic acid A		DSS-induced mice model of UC	Neutrophil recruitment, activation and infiltration↓	TJs (ZO-1, ZO-2, Occludin)↑, MUC-2↑; STAT3/NF-κB↓, p-STAT3↓, p-p65 and p-IκBα↓, pro-inflammatory cytokines (TNF-α, IL-6, IL-1β)↓	Repair the intestinal barrier function; Anti-inflammatory effects	(115)
	Chlorogenic acid		DSS-induced mice model of UC; Apc^min/+^ tumorigenesis mouse models and orthotopic implanted CRC mouse models	4-HPA↓, AK2/STAT3↓, CXCL3↓, PMN-MDSC↓, neutrophil infiltration↓	NF-κB↓, p-P65↓, IL-1β↓, TNFα↓, IL-6↓, IL-12↑; the proportions of Firmicutes and Bacteroidetes↓, the relative abundance of Akkermansia↑, mucus layer thickness↑	Anti-inflammatory effects; Repair the intestinal barrier function; Anti-cancer effects	(116, 117)
	Curcumin		The colonic mucosa in an experimental model of DC; DSS-induced mice model of colitis; TNF-α-induced mice model of colitis	MPO↓, neutrophil recruitment and infiltration↓	NF-κB↓, pro-inflammatory cytokines (TNF-α, IL-6, IL-8)↓, cytokines↓, antioxidant gene↑; iNOS↓, NO↓, ROS↓; Bcl-6-Syk-BLNK↓, the transformation of primordial B cells into memory B cells↑, induced memory B cells/pro-inflammatory factors↓, tolerant memory B cells/anti-inflammatory factors↑, CXCR3↓	Anti-inflammatory effects; Reduce oxidative stress; Modulate immune response	(118–120)
	Astragalin		DSS-induced mice model of UC	Neutrophil activation and infiltration↓	ZO-1↑, Occludin↑, Muc2↑; NF-κB↓, pro-inflammatory cytokines (TNF-α, IL-6, IL-1β)↓; Escherichia-Shigella↓, Ruminococcaceae↑	Repair the intestinal barrier function; Anti-inflammatory effects; Regulate gut microbiota	(121)
	Hesperetin		LPS-indueed septie rats	LC3B↓, LC3-I/LC3-II↓, CitH3↓, MPO↓, NETs↓	I-FABP↓, TJs (Occludin, Claudin-1)↑; TLR4/NF-κB↓, Nrf2↓, HO-1↓, MDA↓, GSH↓, LPO↓, ROS↓	Repair the intestinal mechanical barrier function; Reduce oxidative stress; Reduce autophagy	(122)
	Quercetin		DSS-induced mice model of UC; HCT116 cells-inoculated mouse model of CRC	Ahr↑, Arnt↑, ROS/Nqo1↓, NETs↓; PAD4↓, Cit-H3↓, NETs↓; neutrophil chemotaxis, activation and infiltration↓	Ahr↑, NF-κB↓, pro-inflammatory cytokines↓, TNF/IL-17/AGE-RAGE↓, IL1β↓, IL6↓, CXCL8↓, MMP9↓; Th17 differentiation↓, Tregs differentiation↑; TJs↑; PAD4↓, IEC apoptosis↓	Anti-inflammatory effects; Modulate immune response; Repair the intestinal barrier function; Regulate the “epithelial-neutrophil” interaction	(123–125)
	Dihydromyricetin		DSS-induced mice model of acute colitis	HIF-1α/VEGFA↓, NETs↓	ZO-1↑, Occludin↑	Repair the intestinal barrier function	(126)
	Kurarinone		Trinitrobenzene sulfonic acid (TNBS)-induced mice model of colitis	Neutrophil chemotaxis, activation and infiltration↓	TJs (ZO-1, Ocludin, Claudin, E-cadherin)↑, goblet cell↑, mucins↑; beneficial microbial taxa (Lactobacullus, Lactobacillales and Lactobacillaceae)↑, harmful microbial taxa (Mollicutes)↓; Blimp-1↑, IL-17A↓, IL-10↑, pro-inflammatory cytokines↓	Repair the intestinal barrier function; Regulate gut microbiota; Anti-inflammatory effects	(127)
	Catechins		Rats subjected to splanchnic artery occlusion and reperfusion; neutrophils and human colon cancer cell line SW480	STAT3↓, CXCL8↓, NETs↓; neutrophil infiltration↓	Pro-inflammatory cytokines (TNF-α)↓, ICAM-1/P-selectin↓; MDA↓, ROS↓	Anti-inflammatory effects; Reduce oxidative stress	(128, 129)
	Glabridin		DSS-induced mice model of colitis and colitis-associated cancer	Neutrophil recruitment, activation and infiltration↓	P-STAT3↓, MMPs (MMP1, MMP3)↓, CXC chemokine (CXCL1, CXCL2)↓; E-cadherin↑, EMT↑	Anti-inflammatory effects; Repair the intestinal barrier function	(130)
	Kaempferol		HCT116 cells-inoculated mouse model of CRC; a murine model of high fat diet-induced obesity and gut inflammation	Neutrophil infiltration↓	VEGF/Akt/p38↓, intestinal microvascular endothelial cells↓; IL1β↓, IL6↓, CXCL8↓, MMP9↓, TLR4/NF-κB↓, pro-inflammatory cytokines (MCP-1, TNF-α, IL-6)↓, chemokines (MCP-1)↓; Desulfovibrio/Helicobacter↓, Parasutterella↑	Improve intestinal microcirculation; Anti-inflammatory effects; Regulate gut microbiota	(125, 131)
Glycosides	Ursolic acid		DSS-induced mice model of UC	Neutrophil activation and recruitment↓	MAPKs↓, IL-6/STAT3↓, IL-6↓, NF-κB↓, pro-inflammatory cytokines↓; PI3K↓, innate immune cells↓; AMPK/FOXO↓, ROS↓; intestinal flora abundance↓	Anti-inflammatory effects; Modulate immune response; Reduce oxidative stress; Regulate gut microbiota	(132)
	Glycyrrhizic acid		AOM/DSS-induced mice model of CAC	PAD4↓, dsDNA↓, MPO↓, ROS↓, Cit-H3↓, NETs↓	CD8+T↓, PD-1↓; Occludin↑, ZO-1↑	Modulate immune response; Repair the intestinal barrier function	(133)
	Anemoside B4		TNBS-induced model of colitis in rats	Neutrophil accumulation and migration↓	S100A9↓, TLR4↓, MAPK (p38, JNK, ERK)/NF-κB↓, pro-inflammatory cytokines (IL-1β, IL-6, TNF-α)↓; apoptotic cells↓, Bax/cleaved caspase3↑, Bcl-2↓	Anti-inflammatory effects; Repair the intestinal barrier function	(134)
Terpenoids	Menthol		AA-induced model of colitis in rats	MPO↓, neutrophil activation and infiltration↓	Calprotectin↓, pro-inflammatory cytokines (IL-1, IL-6, IL-23 TNF-α)↓; MDA↓, GSH↑	Anti-inflammatory effects; Reduce oxidative stress	(135)
	Angelica oil		DSS-induced mice model of UC	Neutrophil infiltration↓	S100A8/A9↓, TJs↑; NF-κB↓, pro-inflammatory cytokines (KC (CXCL1), LIX, G-CSF, IL-1α, IL-6, MCP-1)↓	Repair the intestinal barrier function; Anti-inflammatory effects	(136)
	Okanagan LEO		A mouse model of acute colitis caused by Citrobacter rodentium	MIP-2α↓, neutrophil infiltration↓	Pro-inflammatory cytokines (TNF-α, IFN-γ, IL-22)↓, iNOS↓; Firmicutes (segmented filamentous bacteria)↑; Th17↑	Anti-inflammatory effects; Regulate gut microbiota; Modulate immune response	(137)
	Nerolidol		AA-induced model of colitis in rats	Neutrophil migration and infiltration↓	SOD↑, CAT↑, free radical↓, MDA↓, GSH↑; LOX-1/IL-1β↓, TLR4/NF-κB↓, AMPK/Nrf-2/HO-1↓, pro-inflammatory cytokines (IL-1β, IL-6, IL-23)↓	Reduce oxidative stress; Anti-inflammatory effects	(138)
	Tripterygium wilfordii polycoride		DSS-induced mice model of UC	Neutrophil migration and infiltration↓	NADPH oxidases↓, ROS↓; NLRP3 inflammasome↓, ASC↓, caspase-1↓, pro-inflammatory cytokines↓	Reduce oxidative stress; Anti-pyroptotic effects	(139)
Microbiota-derived metabolites	Short-chain fatty acids	Butyrate	DSS-induced mice model of colitis; acute murine C. jejuni-induced enterocolitis; cefoperazone-pretreated mice model	HDAC↓, MPO↓, ROS↓, NETs↓; neutrophil recruitment and infiltration↓	NF-κB↓, MCP-1/IL-6↑, pro-inflammatory cytokine (TNF-α, IL-8)↓, anti-inflammatory cytokines (IL-10, IL-22)↑; TJs (Claudin-3, Occludin, ZO-1)↑, intestinal mucus↑; GPR43↑	Anti-inflammatory effects; Repair the intestinal barrier function; Regulate the “microbiota-neutrophil” interaction	(140–142)
		Acetate	DSS-induced mice model of colitis; VD-deficient animal models	Neutrophil recruitment and infiltration↓; neutrophil↑	HDAC↓, Treg↓, IgA↑; CXCL2↑, CXCR2↓; Cetobacterium↑, acetate↑, IL-22↑	Repair the intestinal barrier function; Regulate the “endothelial - neutrophil” interaction; Regulate gut microbiota	(143, 144)
	Microalgae		Soybean meal-induced zebrafish model	Neutrophil infiltration↓	Globet↑, mucus secretion↑	Repair the intestinal barrier function	(145)
	Altechromone A		TNBS-induced zebrafish model of IBD	Neutrophil recruitment and migration↓	STAT3/NLRP3↓, NF-κB↓, pro-inflammatory cytokines (IL18)↓; NO↓, ROS↓	Anti-inflammatory effects; Reduce oxidative stress	(146)
Natural medicinal materials	Aucklandiae Radix		DSS-induced mice model of UC	Neutrophil↓	PKM2↓, NF-κB/NLRP3↓, Foxp3↓, Treg differentiation↑, Th17 differentiation↓, pro-inflammatory cytokines (IL-6, TNF-α, IL-1β)↓	Anti-inflammatory effects	(147)
	Tiliae Flos		LPS-stimulated human neutrophils model	Neutrophil apoptosis↑	SCFAs↑, NF-κB↓, pro-inflammatory cytokines (TNF-α)↓	Anti-inflammatory effects	(148)
	Phyllanthus niruri Linn		1, 2 DMH-induced model of CRC in rats	Neutrophil activation↓	CTL↑, NK↑, apoptotic cells↑, TNF-α↓, interferon↓	Anti-inflammatory effects	(149)
	Ethanol extract of cicer arietinum		DSS-induced mice model of colitis	Neutrophil activation↓	NF-κB/STAT3↓, IL-6↓, IL-1β↓, TNF-α↓, COX-2↓, iNOS↓	Anti-inflammatory effects	(150)
	Black raspberries		A mouse model of colorectal cancer (Apc^Min/+^)	Neutrophil activation and infiltration↓	FFAR2↑, cAMP-PKA-CREB/Wnt↓, HDAC↓, acetyl-H3↑, the negative regulators (DKK3, SOX17)↑; IL-1β↓, IL-6↓, TNF-α↓	Anti-cancer effects; Anti-inflammatory effects	(151)
	Hydroethanolic extract of fritillariae thunbergii Bulbus		DSS-induced mice model of UC	Neutrophil↓	TJs (ZO-1, Occludin)↑, goblet cell↑; MMP-9↓, pro-inflammatory cytokines and chemokines (IL-1β, IL-8, TGF-β)↓	Repair the intestinal barrier function; Anti-inflammatory effects	(152)
	Ilex rotunda Thunb.		DSS-induced mice model of UC	Neutrophil activation and infiltration↓	TJs (Occludin, Claudin-1, ZO-1)↑; OSM↓, OSMR↓, STAT3/p-STAT3↓, TLR4/NF-κB↓, pro-inflammatory cytokines (IFN-γ, IL-1β, IL-6)↓	Repair the intestinal barrier function; Anti-inflammatory effects	(153)
	Hydroalcoholic extract of Araucaria sp.		TNBS-induced model of colitis in rats	Neutrophil activation and migration↓	NF-κB↓, pro-inflammatory cytokines↓; acidic and neutral mucins↑; lipid peroxidation↓, GST↑, GSH↑, MDA↓	Anti-inflammatory effects; Repair the intestinal barrier function; Reduce oxidative stress	(154)
	Bixa orellana Leaf		AA-induced model of UC in rats	Neutrophil infiltration↓	SOD↑, CAT↑, GSH↑, MDA↓, nitrite↓, cyclooxygenase and lipooxygenase↓, ROS↓	Reduce oxidative stress	(155)
	A phytopharmaceutical combining sage and bitter apple		DSS-induced mice model of colitis	MPO↓, neutrophil↓	TJs (Claudin-1, Claudin-4, ZO-1)↑, goblet cell↑; chemokines (CXCL-1/KC)↓, pro-inflammatory cytokines and chemokines (COX-2, IgA)↓, anti-inflammatory cytokine (IL-10)↑	Repair the intestinal barrier function; Anti-inflammatory effects	(156)
Traditional Chinese medicine compounds	Wu-Mei-Wan		TNBS-induced mice model of colitis	Neutrophil infiltration↓	OGT↑, OGA↓, RIPK3O-GLC Nacylation↑, RIPK3↓, necrosome↓; pro-inflammatory cytokines (IL-1β, IL-6, TNF-α, IFN-γ)↓	Inhibit necroptotic apoptosis; Anti-inflammatory effects	(157)
	Huang Qin Decoction		AOM/DSS-induced mice model of colitis-associated cancer	PAD4↓, NETs↓; neutrophil infiltration↓	ZO-1↑, occludin↑; TNF-α, IL-1β↓	Repair the intestinal barrier function; Anti-inflammatory effects	(158)
	Qu-Yu-Jie-Du Decoction		DSS-induced mice model of colitis	Neutrophil infiltration↓	Nrf2↑, HO-1↑; pro-inflammatory cytokines (IL-1β, IL-6, TNF-α, TGF-β)↓, chemokines (CCL2, CXCL2)↓	Reduce oxidative stress; Anti-inflammatory effects	(159)
	Huanglian Ganjiang Decoction		DSS-induced mice model of acute colitis	Neutrophil↓	TJs (occludin, claudin-1)↑; pro-inflammatory cytokines (IL-6, IL-10, TNF-α, IFN-γ)↓	Repair the intestinal barrier function; Anti-inflammatory effects	(160)
	Pulsatilla decoction		DSS-induced mice model of colitis	PAD4↓, Cit-H3↓, MPO↓, NETs↓; MMP-7↓, neutrophil activation and infiltration↓	IL-6↑, TJs↑, mucus secretion↑; pro-inflammatory cytokines (TNF-α, IL-1β, IL-6)↓, anti-inflammatory cytokines (IL-4, IL-10)↑, chemokines (CXCL1, CXCL2)↓; Ly6G↓, ROS↓	Repair the intestinal barrier function; Anti-inflammatory effects; Reduce oxidative stress	(161, 162)
	Sijunzi Decoction		DSS-induced mice model of UC	NETs↓; neutrophil infiltration↓	Pro-inflammatory cytokines (IL-1β, TNF)↓, anti-inflammatory cytokines↑; ROS↓; MIR200C-3p↑	Anti-inflammatory effects; Reduce oxidative stress; Repair the intestinal barrier function	(163)

#### 4.1.1 Berberine

Berberine (BBR) is a natural isoquinoline alkaloid mainly isolated from *Berberis aquifolium* Pursh, *Berberis vulgaris* L, and *Coptis chinensis* Franch (164). BBR inhibited the nuclear translocation of neutrophil elastase (NE) and the subsequent formation of NETs by inhibiting the interaction of linc00668 with NE, which was highly enriched in IECs derived exosomes, thereby effectively regulating the IECs-neutrophil interaction (86). Additionally, BBR reduced intestinal bacteria β-glucuronidase (GUS)-producing bacteria and decreased SN38G conversion to SN38 (the active metabolite of CPT11), thus indirectly reducing neutrophil overactivation and infiltration (103).

#### 4.1.2 Berbamine

Berbamine (BBM), a bisbenzylisoquinoline alkaloid extracted from Chinese herbal medicine *Berberis vulgaris* L, has been shown to inhibit the formation of NETs after significantly reducing expression of PAD4 in the colon (104). Moreover, BBM attenuated the release of Cit-H3, MPO and NE, which were NETs-associated products (165, 166).

#### 4.1.3 Lycopodium

Lycopodium (LYCO), a member of the *Lycopodiaceae* family, is enriched in alkaloids and triterpenoids. By restoring the endogenous antioxidants or decreasing the levels of IL-1β and IL-23, LYCO diminished the release of ROS, MPO and free radicals (105, 166). Ultimately, the suppression of ROS inhibited NF-κB signaling and attenuated neutrophil-associated inflammation in the intestinal mucosa (105).

#### 4.1.4 Tetramethylpyrazine

Tetramethylpyrazine, a pyrazine alkaloid derived from the traditional Chinese medicine Chuanxiong, has been demonstrated to reduce neutrophil infiltration in intestinal tissue by decreasing MPO activity and improving intestinal microcirculation (106).

#### 4.1.5 Lemairamin (Wgx-50)

Lemairamin (Wgx-50), a hydroxylamine compound extracted from Sichuan pepper, belongs to the isoquinoline alkaloid class. It inhibited AKT signaling, which could cause reduced expression of pro-inflammatory cytokines, comprising IL-1β, IL-6, CXCL8a, and TNF-α. Then, Wgx-50 directly suppressed neutrophil recruitment to sites of intestinal injury (107).

#### 4.1.6 Cavidine

Cavidine, isolated from *Corydalis* impatiens, is an isoquinoline alkaloid. Cavidine enhanced the activities of SOD and GSH to reduce the release of ROS. Furthermore, it inhibited the NF-κB signaling pathway, which was involved in the reduced transcription and release of TNF-α and IL-6, thereby mitigating neutrophil-associated inflammation (108).

### 4.2 Quinones

Quinones are a class of aromatic organic compounds with six carbon atom cyclic diketone structure containing two double bonds in traditional Chinese medicine. According to their chemical structures, they are mainly divided into four types: benzoquinone, naphthoquinone, phenanthraquinone, and anthraquinone. These compounds have significant biological activities, possessing antioxidant, antibacterial, and antitumor properties activities.

#### 4.2.1 Tanshinone IIA

Tanshinone IIA, a phenanthrenequinone compound derived from *Salvia miltiorrhiza*, attenuated the NF-κB signaling pathway and resulted in a reduction in the transcription and release of pro-inflammatory cytokines, then mitigating neutrophil-associated inflammation and ROS release (109).

#### 4.2.2 Rhein

Rhein, an anthraquinone compound derived from rhubarb. It concurrently reduced the production of pro-inflammatory cytokines and cleaved IL-1β by suppressing NF-κB signaling and the assembly of the NLRP3 inflammasome, ultimately exerting anti-pyroptotic effects and mitigating neutrophil-mediated inflammation (110).

### 4.3 Polysaccharides

Polysaccharides, large biomolecules formed by the condensation and dehydration of monosaccharide units, are essential carbohydrates with complex molecular structures. It is important to distinguish between pathogen-derived polysaccharides such as LPS, and the plant-derived or fungal-derived polysaccharides discussed herein. LPS, a unique component of Gram-negative bacterial cell walls, is a potent pro-inflammatory molecule. It induces an inflammatory response by stimulating host cells to produce a large number of inflammatory cytokines (167). In contrast, the polysaccharides from medicinal plants or fungi (e.g., *Atractylodes macrocephalae*, *Grifola frondosa*) typically exhibit a range of physiological functions, comprising antioxidant, immune-modulatory, anti-infective, etc, thereby exerting protective effects in intestinal inflammation.

#### 4.3.1 *Atractylodes macrocephalae* Koidz. and *Grifola frondosa* polysaccharides

Polysaccharide is an effective component of *Atractylodes macrocephalae* Koidz. (AMP), which is a homologous plant of invigorating spleen and replenishing qi. *Grifola frondosa*, a medicinal and edible fungus, contains *Grifola frondosa* polysaccharides (GFPs) as main active components. The polysaccharides from AMP and GFPs directly inhibited neutrophil infiltration, migration and aggregation, together suppressing the levels of inflammatory cytokines in the colonic mucosa (111, 113). Moreover, AMP modulated gut microbiota composition to lower pro-inflammatory signals like LPS, which achieved microbiota-neutrophil interactions (111).

#### 4.3.2 *Agaricus blazei* Murill polysaccharides

*Agaricus blazei* Murill polysaccharides (ABPs) are the principal bioactive constituents of Agaricus blazei fruiting bodies, and ABP1 is the major component in ABPs. ABP1 increased the abundance of SCFA-producing bacteria, and promoted elevated butyrate levels, subsequently impeding neutrophil recruitment and modulating microbiota-neutrophil interactions (112).

### 4.4 Polyphenols

Polyphenols are a class of compounds widely present in natural products with multiple benzene rings and hydroxyl functional groups in their chemical structure, including flavonoids, flavonols, phenolic acids, phenylpropanoids, and anthocyanins. Polyphenols have a variety of pharmacological activities, containing antioxidant, anti-inflammatory, antibacterial and so on.

#### 4.4.1 Forsythiaside A

Forsythiaside A, a phenylethanolic glycoside primarily isolated from *Forsythia suspensa* (Thunb.) Vahl, could downregulate the expression of PAD4 in colon neutrophils. This modulation led to a reduction in the levels of Cit-H3 and MPO, as well as in the expression of pro-inflammatory cytokines. Ultimately, the reduction attenuated the formation of NETs and alleviated colonic neutrophil infiltration (114).

#### 4.4.2 Isochlorogenic acid A

Isochlorogenic acid A (ICGA-A), a dicaffeoylquinic acid, is abundantly present in various medicinal plants and vegetables. It has been shown to inhibit the STAT3/NF-κB pathway, then attenuating neutrophil activation and infiltration (115).

#### 4.4.3 Chlorogenic acid

Chlorogenic acid (ChA) is a natural polyphenol predominantly found in coffee and various other plants. On one hand, it inhibited the NF-κB/p65 to decrease neutrophil infiltration (116, 168). On the other hand, it increased the relative abundance of Akkermansia in the intestine, and improved the integrity of the mucus layer through extracellular vesicles derived from Akkermansia mucosae (169). Concurrently, ChA inhibited the production of 4-HPA, interfered with JAK2/STAT3 signaling, and downregulated CXCL3 transcription, resulting in reduced recruitment of PMN-MDSC in CRC (117).

#### 4.4.4 Curcumin

Curcumin, the bioactive natural polyphenol, is a substance derived from the rhizome of *Curcuma longa*. It reduced MPO activity, and limited the release of ROS (118, 119). Moreover, Curcumin exerted inhibitory effects on the activation of the Bcl-6-Syk-BLNK signaling pathway, promoted the conversion of naive B cells into memory B cells, restored the balance between pro-inflammatory and anti-inflammatory factors, and reduced CXCR3 expression. All contributed to a reduction in neutrophil recruitment to the colon (120, 170).

#### 4.4.5 Astragalin

Astragalin (AG), a natural flavonoid found in *Moringa oleifera*, *Radix astragali*, *Morus alba*, and *Cassia alata* (171), suppressed NF-κB pathway, reduced the expression of pro-inflammatory cytokines, then inhibiting neutrophil infiltration in the colon. In addition, AG attenuated TLR4-mediated signaling, which was modulated by the abundance of intestine bacteria, leading to inhibiting neutrophil activation (121).

#### 4.4.6 Hesperetin

Hesperetin, one of the major flavonoids with multiple biological activities, regulated the TLR4/NF-κB signaling pathway and concurrently inhibited the expression of LC3B, resulting in a reduced LC3-I/LC3-II ratio in neutrophils. In the meantime, Hesperetin decreased NETs formation and Cit-H3 production via the ROS/autophagy pathway (122).

#### 4.4.7 Quercetin

Quercetin (QUE) is a natural flavonoid widely found in fruits and Chinese herbal medicines. Through activating AhR, QUE involved the upregulation of Arnt, reduced ROS and Nqo1 production, which caused an inhibition of NETs formation. Additionally, QUE has been observed to suppress IL-6 expression, decrease Th17 differentiation and neutrophil activation, thus mitigating tissue damage. It mitigated neutrophil-induced IECs apoptosis by inhibiting NF-κB activation and reducing PAD4-mediated NETosis, thereby modulating the detrimental “IECs-neutrophil” crosstalk (123, 124). Furthermore, QUE downregulated the expression of key inflammatory factors (IL1β, CXCL8, and MMP9), inhibiting neutrophil activation and infiltration, as well as suppressing tumor growth in CRC (125).

#### 4.4.8 Dihydromyricetin

Dihydromyricetin, a flavonoid derived from the herb *Ampelopsis grossedentata*, targeted neutrophils and inhibited the HIF-1α/VEGFA signaling pathway, thus reducing the formation of NETs (126).

#### 4.4.9 Kurarinone

Kurarinone, a major flavonoid compound from the dried roots of *Sophora flavescens*, restored gut microbiota and upregulated Blimp-1. These effects decreased IL-17A secretion by Th17 cells and increased IL-10 expression, preventing neutrophil infiltration and reducing neutrophil chemotaxis and activation (127).

#### 4.4.10 Catechins

Green tea and black tea contain polyphenolic compounds, particularly catechins, such as epigallocatechin-3-gallate (EGCG). Catechins reduced TNF-α levels, significantly downregulated ICAM-1 and P-selectin expression, as well as decreased lipid peroxidation products like MDA. These actions inhibited ROS production and reduced neutrophil infiltration (128). EGCG suppressed NETs formation by modulating the STAT3/CXCL8 signaling pathway, afterwards inhibiting the migration and invasion of colorectal cancer (129).

#### 4.4.11 Glabridin

Glabridin is a flavonoids compound isolated from *Glycyrrhiza glabra* L. It inhibited the phosphorylation of the transcription factor STAT3, leading to the downregulation of MMP1 and MMP3 expression. By suppressing MMPs, Glabridin decreased the release of CXCL1 and CXCL2, thus reducing neutrophil migration and infiltration (130).

#### 4.4.12 Kaempferol

Kaempferol, a major flavonol widely found in various fruits and vegetables, downregulated the expression of key inflammatory mediators (IL-1β, CXCL8, and MMP9), then reducing neutrophil infiltration and activation while inhibiting tumor growth in CRC (125). Additionally, Kaempferol decreased TLR4/NF-κB pathway activation, which reduced the expression of chemokines and pro-inflammatory cytokines, subsequently inhibiting neutrophil infiltration in colitis (131).

### 4.5 Glycosides

Glycosides are compounds formed by the glycosidic linkage between the carbon atoms of sugar or sugar derivatives and non-sugar substances, which have antioxidant, anti-inflammatory, anti-cancer and other biological activities. According to the structural types of aglycones, they are most commonly divided into cyanogen glycosides, phenolic glycosides, alcohol glycosides, anthracene glycosides, flavone glycosides, saponins, cardiac glycosides, coumarin glycosides and iridoid glycosides.

#### 4.5.1 Ursolic acid

Ursolic acid (UA), a naturally occurring pentacyclic triterpenoid carboxylic acid, is extracted from various medicinal plants and foods (172). UA has been shown to downregulate the PI3K signaling pathway, and suppress early immune responses triggered by neutrophils via the IL-6/STAT3 signaling pathway. It inhibited the activation of the NF-κB pathway, subsequently inhibiting the recruitment and activation of neutrophils at inflammation sites induced by chemokines. Furthermore, UA reduced ROS generated through activating the AMPK/FOXO signalling pathway in fatty acid metabolism. This multifaceted action alleviated oxidative stress mediated by neutrophils (132).

#### 4.5.2 Glycyrrhizic acid

Glycyrrhizic acid (GA), a natural pentacyclic triterpenoid compound derived from licorice, could significantly reduce dsDNA, MPO-DNA complexes, ROS and Cit-H3 by inhibiting the enzymatic activity of PAD4. This action resulted in a marked decrease in the formation of NETs (133).

#### 4.5.3 Anemoside B4

Anemoside B4 is the predominant triterpenoid saponin isolated from *Pulsatilla chinensis*. It decreased TLR4 activation by inhibiting S100A9 expression, subsequently suppressing the activation of MAPK (p38, JNK, ERK) and NF-κB signaling pathways. The effects resulted in suppressing the migration and accumulation of neutrophils in the colon directly or indirectly (134).

### 4.6 Terpenoids

Terpenoids are a general term for all polymers of isoprene and their derivatives, with a general formula (C5H8)n. In addition to existing as terpene hydrocarbons, they also form a wide variety of oxygen-containing derivatives. Terpenoids have diverse pharmacological activities, such as antibacterial, antitumor, anti-inflammatory, and coronary artery dilation.

#### 4.6.1 Menthol and nerolidol

Menthol, a monocyclic monoterpene, serves as the primary active ingredient in the peppermint plant, while nerolidol is a naturally occurring aliphatic sesquiterpene alcohol. Both compounds have been demonstrated to reduce MPO activity, inhibit neutrophil activation, and diminish infiltration by enhancing tissue levels of SOD and CAT, reducing the formation of lipid peroxides and MDA, and restoring GSH bioavailability (135, 138).

#### 4.6.2 Angelica oil

Volatile Angelica oil (AO) has been identified as a significant constituent of Angelica sinensis. AO mitigated the activation of the TLR4/NF-κB pathway, resulting in a reduction in cytokine production, simultaneously suppressing neutrophil infiltration in the colon (136).

#### 4.6.3 Okanagan lavender essential oil

A unique blend of essential oils derived from lavender is referred to as Okanagan Lavender Essential Oil (OLEO). OLEO inhibited MIP-2α-mediated neutrophil infiltration into intestinal crypts (137).

#### 4.6.4 *Tripterygium wilfordii* polycoride

*Tripterygium wilfordii* polycoride (TWP), the primary active compound of *Tripterygium wilfordii*, consists predominantly of diterpenoid and triterpenoid compounds. TWP has been demonstrated to significantly decrease NADPH oxidase activity and ROS production. It inhibited the activation of NLRP3 inflammasome, ASC and caspase-1, which ultimately reduced neutrophil infiltration via the inhibition of pyroptosis (139).

### 4.7 Microbiota-derived metabolites

#### 4.7.1 Short-chain fatty acids

Short-chain fatty acids (SCFAs), major metabolites derived from gut microbiota, play a beneficial role in gastrointestinal health. The principal SCFAs, including acetate, propionate and butyrate, constitute more than 95% of the total SCFA content in feces.

##### 4.7.1.1 Butyrate

By downregulating HDAC activity, butyrate reduced the release of MPO and ROS to inhibit the formation of NETs, and suppressed neutrophil migration induced by IL-8 (140). Meanwhile, butyrate suppressed NF-κB signaling pathway and the secretion of pro-inflammatory cytokines such as TNF-α with increased levels of MCP-1 and IL-6. These combined actions culminated in reduced neutrophil infiltration (141). For one thing, butyrate could induce neutrophil recruitment through the direct signaling of GPR43, for another, it could indirectly regulate neutrophils through the activation of other types of immune cells (142).

##### 4.7.1.2 Acetate

Acetate upregulated CXCL2 protein expression and downregulated its receptor CXCR2 on colonic neutrophils, thereby inhibiting neutrophil recruitment and infiltration (143). It also delayed neutrophil apoptosis through FABP4 (110). Moreover, vitamin D encouraged the proliferation of acetate-producing bacteria, particularly *Cetobacterium*. The resulting increase in acetate enhanced IL-22 expression, which in turn stimulated neutrophil production (144).

#### 4.7.2 Microalgae

Microalgae represent a diverse group of microorganisms that include diatoms, dinoflagellates, and flagellates. Microalgae reduced soybean meal-induced neutrophil infiltration by increasing goblet cell numbers and enhancing mucus secretion (145).

#### 4.7.3 Altechromone A

Altechromone A could be isolated from the marine-derived fungus *Penicillium chrysogenum* (173). This compound inhibited both the STAT3 and NLRP3 pathways. This led to decreased expression of pro-inflammatory genes and reduced release of NO and ROS, resulting in limited neutrophil migration and accumulation (146).

### 4.8 Natural medicinal materials

#### 4.8.1 Aucklandiae Radix

In Chinese Pharmacopoeia, costunolide is the principal active constituent of Aucklandiae Radix. It promoted the differentiation of Treg cells and reduced the differentiation of Th17 cells through downregulating PKM2 expression, which subsequently diminished the accumulation of neutrophils in the intestinal tract (147).

#### 4.8.2 Tiliae Flos

The pharmacological activity of *Tiliae Flos* is primarily attributed to its flavonoid, saponin, and phenolic acid constituents. Its extract has been shown to elevate SCFA levels, inhibit NF-κB signaling, and suppress TNF-α production, inducing neutrophil apoptosis (148).

#### 4.8.3 Phyllanthus niruri Linn

*Phyllanthus niruri Linn* comprises active phytochemicals in various parts, including flavonoids, alkaloids, terpenoids, lignin, polyphenols, tannins, coumarin and saponins. It increased the expression of cytotoxic T lymphocytes and NK cells, also elevated the number of apoptotic cells to reduce respiratory burst and the release of TNF-α and interferon-induced proteins. Collectively, the process finally inhibited the pro-inflammatory functions of intestinal neutrophils (149).

#### 4.8.4 *Cicer arietinum* L.

*Cicer arietinum* L. (CEE) is known for its high lysine content. The ethanol extract of CEE inhibited NF-κB/STAT3 signaling pathways, causing reduced expression of IL-6, IL-1β, TNF-α, COX-2 and iNOS, and ultimately inhibiting neutrophil activation (150).

#### 4.8.5 Black raspberries

Black raspberries are rich in soluble fiber, which undergoes colonic fermentation to produce SCFAs. It upregulated FFAR2 expression and inhibited both the cAMP-PKA-CREB and Wnt signaling pathways, resulting in decreased infiltration of GR-1^+^ neutrophils within intestinal polyps (151).

#### 4.8.6 *Fritillariae thunbergii* Bulbus

Phytochemical investigations of *Fritillariae thunbergii* Bulbus (FTB) have identified several bioactive alkaloids, containing sipeimine, peiminine, and yibeissine. Hydroethanolic extract of FTB decreased neutrophil infiltration into intestinal tissues and modulated extracellular matrix remodeling by suppressing MMP-9 activity during inflammation (152).

#### 4.8.7 *Ilex rotunda* Thunb.

Ziyuglycoside I, Ziyuglycoside II, syringin, and pedunculoside are the main active components of *Ilex rotunda* Thunb. (IR). The primary active components downregulated the expression of OSM and OSMR proteins, inhibiting the activation of STAT3 pathway. IR also suppressed the TLR4/NF-κB signaling pathway, thus mitigating neutrophil activation and infiltration (153).

#### 4.8.8 Hydroalcoholic extract of *Araucaria* sp.

*Araucaria* sp. is a hypropolis species particularly rich in labdane diterpenes. Its hydroalcoholic extract attenuated oxidative stress by reducing lipid peroxidation and preserving GST enzyme activity, along with inhibiting MPO activity and chemotactic migration, thereby leading to a significant reduction in neutrophil numbers (154).

#### 4.8.9 *Bixa orellana* leaf hydroethanolic extract

The hydroethanolic extract of *Bixa orellana* modulated oxidative stress through the direct scavenging of ROS or inhibition of cyclooxygenase and lipoxygenase enzymes, contributing to a reduction in neutrophil infiltration (155).

#### 4.8.10 A phytopharmaceutical combining sage and bitter apple

A phytopharmaceutical combining sage and bitter apple reduced the expression of neutrophil chemokine CXCL1/KC in colon tissue. It also inhibited MPO activity and the expression of pro-inflammatory cytokines and chemokines, promoted expression of the anti-inflammatory cytokines, which further mitigated neutrophil infiltration (156).

### 4.9 Traditional Chinese medicine compounds

#### 4.9.1 Wu-Mei-Wan

Wu-Mei-Wan formula reduced RIPK3 activation, necrosome formation, and the levels of IL-1β, IL-6, TNF-α and IFN-γ to inhibit neutrophil necrotic apoptosis and neutrophil infiltration (157).

#### 4.9.2 Huang Qin decoction

Huang Qin Decoction inhibited the formation of NETs by downregulating the expression of PAD4. Besides, it alleviated neutrophil infiltration in the colon caused by TNF-α and IL-1β (158).

#### 4.9.3 Qu-Yu-Jie-Du decoction

Qu-Yu-Jie-Du Decoction downregulated the expression of CCL2 and CXCL2 in colon tissues, then reducing local neutrophil infiltration (159).

#### 4.9.4 Huanglian Ganjiang decoction

Alkaloids such as berberine, berberrubine, oxyberberine are the main ingredients in cold-natured medicine CP extracts in Huanglian Ganjiang Decoction. By contrast, volatile oil is the main active constituent in hot-natured medicine AZ. CP reduced production of pro-inflammatory cytokines and neutrophil infiltration while AZ exerted advantages in regulating neutrophils by enhancing the production of anti-inflammatory immune cells and cytokines (160).

#### 4.9.5 Pulsatilla decoction

Pulsatilla decoction (PD) reduced MMP-7 expression and decreased neutrophil infiltration to suppress intestinal inflammation (161). Simultaneously, the n-butanol extract of PD reduced the levels of chemokines CXCL1 and CXCL2, inhibited Ly6G expression and ROS generation, effectively decreasing neutrophil infiltration and activation. It led to the suppression of key proteins involved in NETs formation (MPO, PAD4, Cit-H3), resulting in diminished NETs release (162).

#### 4.9.6 Sijunzi decoction

The practical components of Sijunzi Decoction (SJZ) may be ginsenoside Rh2, isoflavones and formononetin. SJZ targeted IL1β and TNF to decrease ROS production and reduced intestinal NETs formation. Meanwhile, it upregulated MIR200C-3p, enhancing intestinal mucosal barrier function and reducing neutrophil infiltration (163).

### 4.10 Additional information

To facilitate a systematic comparison of therapeutic effects, the aforementioned natural products are categorized according to their respective mechanisms of action (**Supplementary Table 1**). The classification is based primarily on neutrophil-related functional regulatory pathways, including ROS production, NETs formation, degranulation, secretion of inflammatory factors (thereby stimulating other immune cells), neutrophil apoptosis or pyroptosis, chemotaxis, tissue infiltration, and recruitment. Furthermore, experimental data from multiple models involving these natural products have been consolidated (**Supplementary Table 2**), revealing their dose–response relationships and toxicity profiles. Natural products typically exhibit multi-target and synergistic regulatory features, with ROS, NETs, and the NF-κB pathway representing the core regulatory mechanisms involved. In contrast, research on other regulatory pathways remains relatively limited. The collected studies primarily consisted of *in vivo* animal experiments using various administration routes, among which oral administration was the most common. Nevertheless, comprehensive safety evaluations—particularly detailed toxicity profiles—are still lacking for most of these natural products, highlighting a significant gap that warrants further investigation. The compiled information is expected to provide a valuable reference for future drug development and mechanistic studies.

## 5 Novel therapeutic strategies targeting neutrophils

### 5.1 Based on nanotechnology

#### 5.1.1 Nanomedicine delivery systems

**Figure 4:**
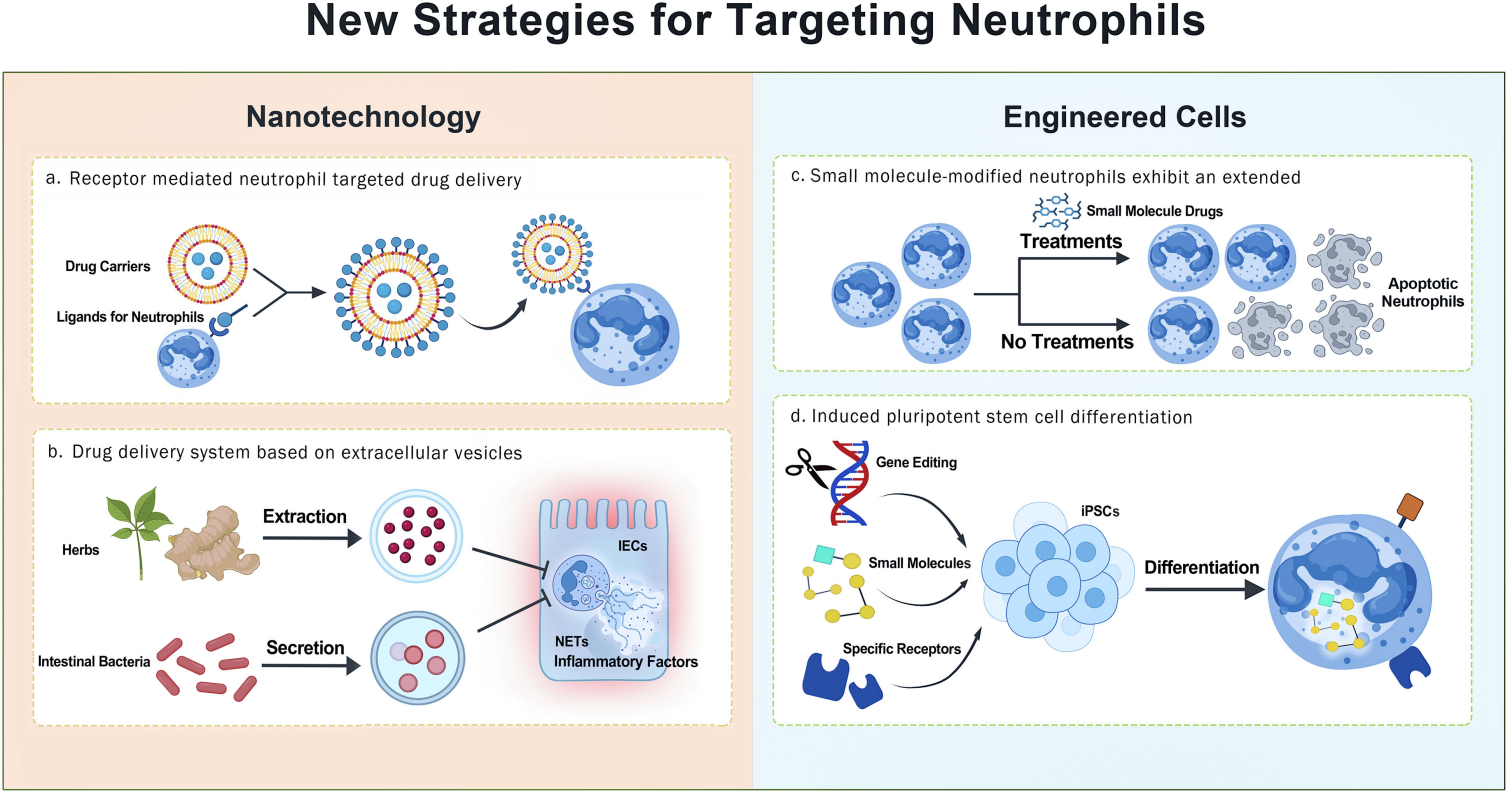
The figure illustrates the latest methods of regulating neutrophil function through nanotechnology and engineered cell strategies.

Neutrophils have been widely used in developing various drug-delivery systems because they can quickly respond to inflammatory signals, migrate across the endothelium, and penetrate deep tissues (174, 175). In recent years, neutrophil-targeted nanodrug precise delivery systems have emerged. Notably, promising results have been observed in models of IBD and CRC (**Figure 4**).

**Receptor-mediated neutrophil targeting**.

Another strategy is receptor-mediated targeting. By modifying the surface of drug carriers with ligands that bind to specific markers on neutrophils, drugs can be directly delivered to neutrophils in circulation or at sites of inflammation, thereby exploiting neutrophils for drug transport (176). The strategy demonstrated that targeting neutrophil receptors or enzymes can significantly enhance delivery precision. Drugs are released only around activated neutrophils under disease conditions, thereby avoiding broad immunosuppression of resting neutrophils.

**Extracellular vesicles**.

Extracellular vesicles (EVs) are nanoscale vesicles secreted by cells that can carry proteins, lipids, and nucleic acids, playing crucial roles in intercellular communication and immune regulation (177). EVs, owing to their stability and biocompatibility, can enhance the stability of natural products and prevent their rapid degradation *in vivo* (177). A typical example is exosome-like nanoparticles derived from Coptidis chinensis (Cc-ELNs). In a DSS-induced colitis mouse model, Cc-ELNs selectively accumulated at inflammatory sites, significantly reduced neutrophil infiltration, and effectively inhibited NETs formation by delivering abundant miR-5106 to downregulate Slc39a2 expression and restore zinc homeostasis in neutrophils (178). In addition, Yang Yi et al. reported that outer membrane vesicles secreted by the gut commensal Bacteroides fragilis can be taken up by neutrophils. By delivering miR-5119, they downregulate PD-L1 expression, which inhibits GSDMD-mediated NETs formation, ultimately alleviating DSS-induced colitis symptoms and promoting intestinal stem cell repair (179). This finding revealed a unique mechanism by which microbe-derived vesicles intervene in inflammation through the regulation of NETs formation.

**Other nanodrug delivery systems**.

As research progresses, an increasing number of nanodrug delivery systems have been developed, including self-assembled nanodelivery systems of natural products, nanosuspensions, nanoliposomes, polymeric micelles, microemulsions/self-emulsifying systems, and solid lipid nanoparticles (180, 181). Importantly, many of these nanosystems exhibit preferential accumulation in inflamed intestinal tissues.

As an example, during decoction, the traditional Chinese medicine formula QY305 can self-assemble into nanoscale subunit structures termed N-QY305, which could inhibit neutrophil chemotaxis toward CXCL2 in colonic tissues (180). Similarly, binary self-assembly of curcumin and glycyrrhizic acid into nanospheres, when incorporated into an inulin hydrogel for oral administration, exhibits favorable gel formation and colon enzyme-triggered drug release (182). Although the application of these technologies in neutrophil-targeted therapy is still nascent, integrating natural product-based nanodelivery systems with neutrophil-targeted carriers represents a promising future direction for the treatment of intestinal disorders such as IBD and even CRC (181).

#### 5.1.2 Other nanotechnologies

Beyond conventional nanodrug delivery, emerging nanotechnology strategies aimed at clearing NETs show therapeutic potential. While DNase I can degrade NETs and alleviate intestinal inflammation, its clinical utility is limited by intrinsic instability and rapid clearance. To overcome these limitations, Wang et al. developed a stable DNase nanozyme (DNase-NZ) that retained prolonged enzymatic activity and exerted superior efficacy in colitis mice compared to free DNase (183). In a similar vein, Dong et al. designed an oral delivery system for staphylococcal nuclease (SNase) that enabled localized NETs degradation in the colon and promoted mucosal repair (184). These advances underscore the promise of nanotechnology-enabled NETs clearance as an adjunctive therapy for intestinal immune disorders.

### 5.2 Engineered cell drug delivery

Engineered neutrophil-based drug delivery leverages multidisciplinary strategies to transform these cells into sophisticated therapeutic carriers. Key innovations are as follows: Genetic engineering, exemplified by generating chimeric antigen receptor-expressing neutrophils from human-induced pluripotent stem cells via CRISPR/Cas9, enable specific tumor targeting without provoking inflammation (185). Chemical and surface modification, where small molecules or conjugated functional groups are used to extend neutrophil lifespan and enhance targeting precision (186, 187). Functional mimicry, such as introducing artificial receptors like hM4Di into myeloid cells to bestow chemotactic responsiveness to inert ligands, thereby augmenting directional migration and bacterial phagocytosis (188). By integrating active targeting, programmable drug release, and intrinsic immune functions, these engineered or neutrophil-mimetic cells achieved amplified specificity, reduced off-target effects, and improved therapeutic efficacy compared with conventional nanocarriers.

## 6 Discussion

This review has systematically summarized the compelling evidence that numerous natural products can precisely modulate the plasticity and function of intestinal neutrophils, thereby ameliorating inflammation and tissue damage in conditions such as IBD and CRC. The extraction of alkaloids, quinones, polysaccharides, polyphenols, glycosides, terpenoids, microbiota-derived metabolites compounds from natural sources has been investigated. We detail their multi-targeted influences on key neutrophil processes, encompassing migration, NETs formation, cytokine release, and oxidative metabolism. Furthermore, we have discussed the underlying molecular mechanisms behind these immunomodulatory actions, as well as the potential of emerging neutrophil-targeted strategies, to enhance the delivery and efficacy of natural products.

The bidirectional regulation of neutrophil function by natural compounds still faces multifaceted challenges in translating from basic biological research to clinical applications. Despite recent advances in single-cell sequencing that have identified several functionally distinct neutrophil subsets in the intestinal mucosa, our understanding of neutrophil heterogeneity remains incomplete, particularly with regard to the dynamic mechanisms and regulatory circuits governing their phenotypic plasticity under different disease contexts (10, 43). Current data derive primarily from limited disease models and tissue sources, necessitating more comprehensive single-cell analyses across diverse intestinal immune disorders. The precise relationship between circulation and tissue-resident intestinal neutrophils remains inadequately defined, with insufficient evidence on their trafficking dynamics and functional transitions. Moreover, the regulatory mechanisms governing neutrophil maturation and phenotypic specialization within the intestinal microenvironment require further investigation.Additionally, several challenges remain. The lack of clear definitions of bioavailability, metabolic conversion, effective tissue concentration, and precise molecular targets hinders their clinical translation.

Nevertheless, natural product research targeting intestinal neutrophils remains relatively conservative and limited in scope. The therapeutic mode of action for natural products is characterized by multi-target network regulation, yet the systematic mapping of their mechanism networks remains in its infancy (189). Indeed, research primarily focuses on superficial interactions between the gut microbiota, the epithelial, and neutrophils, with restricted exploration of interactions involving other immune cells. Next, therapeutic mechanisms continue to be narrowly concentrated on key targets such as NETs, NF-κB inflammatory cascades, pyroptosis and oxidative stress. Lastly, research on novel delivery methods for natural products targeting neutrophils remains scarce. In current studies, the common approach is to use conventional oral or injectable administration routes, and there is little exploration of innovative delivery methods like nanocarriers and targeted delivery systems for regulating neutrophils with natural products. The status quo imposes limitations on the potential of natural products in the treatment of intestinal immune disorders. It is imperative that further research be conducted on innovative delivery methods with a view to broadening their clinical application prospects.

Looking forward, future research should focus on several key directions. First, a deeper understanding of the molecular mechanisms governing neutrophil heterogeneity and natural product interactions is essential. Mechanistic investigations must advance beyond phenomenological observations to elucidate the molecular determinants of neutrophil heterogeneity and the precise interaction networks through which natural products exert their immunomodulatory effects (10, 43). Second, the integration of compounds with cutting-edge biotechnologies faces inherent challenges, including inherent heterogeneity, lack of standardized separation and characterization protocols, unclear *in vivo* pharmacokinetics, and obstacles in scalable production and storage stability (190, 191). Advanced delivery systems should be optimized to enhance the specificity and accuracy of drug delivery to the intestinal neutrophil, and to ensure functionality, sustained safety and stability (183, 184). Third, rigorous preclinical and clinical studies are needed to validate the efficacy and safety of natural products, either as monotherapies or in combination with conventional treatments (192, 193). Collectively, natural products represent a valuable resource for developing next-generation neutrophil-targeted therapies. These suggestions might usher in a new paradigm of developing novel therapies targeting intestinal neutrophils for patients.

## 7 Conclusion

This review summarizes the heterogeneity and plasticity of intestinal neutrophils and their dynamic interactions with the microbiota, epithelial barrier, and other immune cells. We compile current evidence demonstrating how natural products modulate neutrophil activation, migration, NETs formation, inflammatory signaling, pyroptosis, and oxidative stress. We further evaluate the therapeutic efficacy and safety of natural products, and highlight emerging neutrophil-targeted technologies to enhance the delivery and efficacy. Collectively, these findings highlight the significant potential of natural products as integrative immunomodulatory interventions targeting intestinal neutrophils.
