# Supplementary material for: Targeting the plasticity of intestinal neutrophils: bidirectional regulation strategies by natural products
Source: Front Immunol. 2026 Jan 12;16:1754107. doi: 10.3389/fimmu.2025.1754107 (PMC12833395; doi:10.3389/fimmu.2025.1754107)
Supplement: Supplementary file 1 [file DataSheet1.docx]

**Supplementary Table 1**

Natural products are categorized according to their respective mechanisms of action in the gut neutrophil

| **Regulation of neutrophil functions** | **Pathways** | **Natural products** | **References** |
| --- | --- | --- | --- |
| Neutrophil apoptosis / Pyroptosis | ROS/autophagy | Hesperetin | (122) |
|  | NLRP3↓ | Rhein | (110) |
|  | NLRP3/caspase-1↓ | Tripterygium wilfordii polycoride | (139) |
|  | RIPK3↓, Necrosome↓, OGT↑, OGA↓ | Wu-Mei-Wan | (157) |
| Neutrophil chemotaxis | STAT3↓, MMPs (MMP1, MMP3)↓ | Glabridin | (130) |
|  | MIP-2α↓ | Okanagan LEO | (137) |
|  | CXCL-1/KC↓ | A phytopharmaceutical combining sage and bitter apple | (156) |
| Neutrophil infiltration | ICAM-1/P-selectin↓ | Catechins | (128) |
|  | FFAR2↑, cAMP-PKA-CREB/Wnt↓ | Black raspberries | (151) |
|  | CCL2, CXCL2↓ | Qu-Yu-Jie-Du Decoction | (159) |
|  | MMP-7↓ | Pulsatilla decoction | (161) |
| Neutrophil recruitment | IL-1β, TNF-α, IL-6↓ | Berbamine  Cavidine | (104) |
|  |  |  | (108) |
|  | GPR43↑ | Butyrate | (142) |
|  | CXCL2↑, CXCR2↓ | Acetate | (143) |
| ROS | GSH↑, SOD↑, MDA↓ | Lycopodium  Cavidine  Menthol  Nerolidol  Catechins  Bixa orellana Leaf | (105) |
|  |  |  | (108) |
|  |  |  | (135) |
|  |  |  | (138) |
|  |  |  | (128) |
|  |  |  | (155) |
|  | SCFA producing bacteria/butyrate↑ | Agaricus blazei Murill polysaccharides | (112) |
|  | NF-κB↓ | TanshinoneIIA  Curcumin  Hydroalcoholic extract of Araucaria sp. | (109) |
|  |  |  | (118, 119) |
|  |  |  | (154) |
|  | TLR4/NF-κB↓, Nrf2↓, MDA↓, GSH↓ | Hesperetin | (122) |
|  | AMPK/FOXO↓ | Ursolic acid | (132) |
|  | NADPH oxidases↓ | Tripterygium wilfordii polycoride | (139) |
|  | STAT3/NLRP3↓, NF-κB↓ | Altechromone A | (146) |
|  | Nrf2↑, HO-1↑ | Qu-Yu-Jie-Du Decoction | (159) |
| NETs | PAD4↓ | Berbamine  Forsythiaside A  Quercetin  Glycyrrhizic acid  Huang Qin Decoction  Pulsatilla decoction | (104) |
|  |  |  | (114) |
|  |  |  | (123-125) |
|  |  |  | (133) |
|  |  |  | (158) |
|  |  |  | (161, 162) |
|  | LC3B↓, LC3-I/LC3-II↓ | Hesperetin | (122) |
|  | Ahr↑, Arnt↑, ROS/Nqo1↓ | Quercetin | (123) |
|  | HIF-1α/VEGFA↓ | Dihydromyricetin | (126) |
|  | STAT3/CXCL8↓ | Catechins | (129) |
|  | HDAC↓, ROS↓ | Butyrate | (140) |
|  | IL-1β↓, TNF↓ | Sijunzi Decoction | (163) |
| Inflammatory factor（thereby stimulating other immune cells） | Akt↓ | Lemairamin (Wgx-50) | (107) |
|  | NLRP3↓ | Agaricus blazei Murill polysaccharides | (112) |
|  | NF-κB↓ | Lycopodium | (105) |
|  |  | Cavidine | (108) |
|  |  | TanshinoneIIA | (109) |
|  |  | Rhein | (110) |
|  |  | Chlorogenic acid | (116, 117) |
|  |  | Ursolic acid | (132) |
|  |  | Butyrate | (141) |
|  |  | Tiliae Flos | (148) |
|  |  | Hydroalcoholic extract of Araucaria sp. | (154) |
|  | STAT3/NF-κB↓ | Isochlorogenic acid A  Ethanol extract of cicer arietinum | (115) |
|  |  |  | (150) |
|  | Bcl-6-Syk-BLNK↓ | Curcumin | (120) |
|  | TLR4/NF-κB↓ | Astragalin  Angelica oil  Kaempferol  Anemoside B4 | (121) |
|  |  |  | (136) |
|  |  |  | (131) |
|  |  |  | (134) |
|  | Ahr/NF-κB↓ | Quercetin | (123-125) |
|  | Blimp-1↑ | Kurarinone | (127) |
|  | IL-6/STAT3↓ | Ursolic acid | (132) |
|  | STAT3/NLRP3↓, NF-κB↓ | Altechromone A | (146) |
|  | PKM2↓, NF-κB/NLRP3↓ | Aucklandiae Radix | (147) |
|  | CTL (Cytotoxic T Lymphocyte)↑, NK↑ | Phyllanthus niruri Linn | (149) |
|  | MMP-9↓ | Hydroethanolic extract of fritillariae thunbergii Bulbus | (152) |
|  | OSM↓, OSMR↓, STAT3/p-STAT3↓, TLR4/NF-κB↓ | Ilex rotunda Thunb. | (153) |
| Degranulation | The interaction of LINC00668 with , the nuclear translocation of Neutrophil Elastase (NE)↓ | Berberine | (86) |
|  | MPO↓ | Lycopodium  Tetramethylpyrazine  TanshinoneIIA  Curcumin  Menthol  A phytopharmaceutical combining sage and bitter apple | (166, 105) |
|  |  |  | (106) |
|  |  |  | (109) |
|  |  |  | (118, 119) |
|  |  |  | (135) |
|  |  |  | (156) |

**Supplementary table 2**

Dose-response relationships and toxicity profiles of natural products

| **Natural products** | | **Model** | **Disease/Research model** | **In vivo**  **/vitro** | **Administration method** | **Dosage** | **IC50/EC50** | **Toxicity profile** | **References** |
| --- | --- | --- | --- | --- | --- | --- | --- | --- | --- |
| Alkaloids | Berberine | Mice | CPT11‐induced Mice model of intestinal mucositis; Dextran sulphate sodium (DSS)-induced Mice model of IBD | In vivo | Oral gavage; intraperitoneal injection | 50 mg/kg; 1 mg/kg | - | LD50 (intraperitoneal administration)-57.6103 mg/kg; LD50 (intravenous injection)-9.0386 g/kg. | (86, 103, 195) |
|  | Berbamine | Mice | DSS-induced Mice model of UC | In vivo | Oral gavage | 10 and 20 mg/kg | - | - | (104) |
|  | Lycopodium | Rats | Acetic acid (AA)-induced model of IBD in Rats | In vivo | Oral gavage | 50 mg/kg | - | - | (105) |
|  | Tetramethylpyrazine | Rats | Sodium taurocholate-induced ANP model in Rats | In vivo | Femoral vein infusion | 6 g/L | - | LD50 (intravenous injection)-239mg/kg | (106) |
|  | Lemairamin (Wgx-50) | Zebrafish | DSS immersion to establish a Zebrafish colitis model | In vivo | drinking freely | 10 μL/L | - | - | (107) |
|  | Cavidine | Rats | AA-induced model of UC in Rats | In vivo | Oral gavage | 1, 5 and 10 mg/kg | - | - | (108) |
| Quinones | TanshinoneIIA | Mice | Azoxymethane (AOM)/DSS-induced murine CRC model | In vivo | Intraperitoneal injection | 200 mg/kg | - | NOAEL (No Observed Adverse Effect Level)  (injection)-5.76 g/kg/day（Rats） | (109, 196) |
|  | Rhein | Zebrafish; mouse cells | Tail-cutting-induced Zebrafish inflammatory models | In vivo and in vitro | drinking freely | 1, 5 and 20 μM | 0.1 mM | - | (110) |
| Polysaccharides | Grifola frondosa polysaccharides | Mice | An oxazolone-induced Mice model of UC | In vivo | Oral gavage | 80, 160 and 320 mg/kg | - | - | (113) |
|  | A. macrocephalae polysaccharides (AMP) | Mice | DSS-induced Mice model of acute colitis | In vivo | Gastric lavage | 100 mg/kg | - | NOAEL-400 mg/kg;  LOAEL (Lowest Observed Adverse Effect Level)-4000 mg/kg  (Largemouth Bass) | (111, 197) |
|  | Agaricus blazei Murill | Mice | Temporary superior mesenteric artery occlusion-induced mouse intestinal I/R model | In vivo | Oral administration | 10 and 100 mg/kg | - | - | (112) |
| Polyphenols | Forsythiaside A | Mice | DSS-induced Mice model of UC | In vivo | Oral administration | 15, 30 and 60 mg/kg | 1.4 μg/mL | LD50-1.98g/kg | (114, 198, 199) |
|  | Isochlorogenic acid A | Mice; cell lines | DSS-induced Mice model of UC | In vivo and in vitro | Oral administration | 25, 50 and 100 mg/kg; 0, 12.5, 25, 50, 100, 200 μM | - | - | (115) |
|  | Chlorogenic acid | Mice | DSS-induced Mice model of UC; Apcmin/+ tumorigenesis mouse models and orthotopic implanted CRC mouse models | In vivo | Oral administration | 1 mM | - | - | (116, 117) |
|  | Curcumin | Mice | The colonic mucosa in an experimental model of DC; DSS-induced Mice model of colitis; TNF-α-induced Mice model of colitis | In vivo | Enema administration; oral administration; intraperitoneal injection | 50 and 200 mg/kg; 100 mg/kg; 25 mg/kg | 6.01 μM | ADI-0-3 mg/kg bw/day; Demethylcurcumin (DC):  LD50 (oral) > 5000 mg/kg (Rats); Acute dermal > 2000 mg/kg  (Rabbits) | (118-120, 200, 201) |
|  | Astragalin | Mice | DSS-induced Mice model of UC | In vivo | Oral gavage | 50, 75 and 100 mg/kg | > 50 μg/mL | - | (121, 202) |
|  | Hesperetin | Rats | LPS-indueed septie Rats | In vivo | Intraperitoneal injection | 50 mg/kg | - | - | (122) |
|  | Quercetin | Mice; cell lines | DSS-induced Mice model of UC; HCT116 cells-inoculated mouse model of CRC | In vivo and in vitro | Oral administration | 20 and 50 mg/kg; 50 mg/kg; 0, 20, 40, 80, 160, 320 µM | 83.47 µM | - | (123-125) |
|  | Dihydromyricetin | Mice | DSS-induced Mice model of acute colitis | In vivo | Oral administration | 50 and 100 mg/kg | - | - | (126) |
|  | Kurarinone | Mice | Trinitrobenzene sulfonic acid (TNBS)-induced Mice model of colitis | In vivo | Intraperitoneal injection | 125 mg/kg | - | - | (127) |
|  | Catechins | Rats; cell lines | Rats subjected to splanchnic artery occlusion and reperfusion; neutrophils and human colon cancer cell line SW480 | In vivo and in vitro | Intravenous injection | 10 and 20 mg/kg; 0, 5, 10, 25, 50 μM | - | - | (128, 129) |
|  | Glabridin | Mice | DSS-induced Mice model of colitis and colitis-associated cancer | In vivo | Oral gavage | 10 and 50 mg/kg | - | LD50 (intraperitoneal) > 400 mg/kg. | (130, 203) |
|  | Kaempferol | Mice; cell lines | HCT116 cells-inoculated mouse model of CRC; a murine model of high fat diet-induced obesity and gut inflammation | In vivo and in vitro | Oral administration | 0.1%; 0, 5, 10, 20, 40, 80 µM | 64.04 µM | - | (125, 131) |
| Glycosides | Ursolic acid | Mice | DSS-induced Mice model of UC | In vivo | Oral gavage | 200 mg/kg | 2.79 μg/mL | LD50 (oral) > 2000 mg/kg; Maximal Tolerable Dose (human): 98 mg/m² | (132, 204-206) |
|  | Glycyrrhizic acid | Mice; cell lines | AOM/DSS-induced Mice model of CAC | In vivo and in vitro | Oral administration | 10, 20 and 30 mg/kg; 20 μM, 40 μM, 60 μM, 80 μM | - | LD50 (oral)-610 mg/kg; LD50 (intraperitoneal injection)-308 mg/kg. | (133, 207) |
|  | Anemoside B4 | Rats; cell lines | TNBS-induced model of colitis in Rats | In vivo and in vitro | Intraperitoneal injection | 5 and 10 mg/kg; 25, 100 and 400 μM | - | LD50 (intravenous injection)-3.36 g/kg. | (134, 208) |
| Terpenoids | Menthol | Rats | AA-induced model of colitis in Rats | In vivo | Oral administration | 50 mg/kg | - | - | (135) |
|  | Nerolidol | Rats | AA-induced model of colitis in Rats | In vivo | Oral administration | 50 mg/kg | - | - | (138) |
|  | Angelica oil | Mice; cells | DSS-induced Mice model of UC | In vivo and in vitro | Oral administration | 10, 20 and 40 mg/kg; 1.56–200 μg/ml | - | - | (136) |
|  | Okanagan LEO | Mice | A mouse model of acute colitis caused by Citrobacter rodentium | In vivo | Oral administration | 50 mg/kg | - | - | (137) |
|  | Tripterygium wilfordii polycoride | Mice | DSS-induced Mice model of UC | In vivo | Oral gavage | 9.01, 27.03 and 81.09 mg/kg | - | - | (139) |
| Microbiota-derived metabolites | Butyrate | Mice; human cells | DSS-induced Mice model of colitis; acute murine C. jejuni-induced enterocolitis; cefoperazone-pretreated Mice model | In vivo and in vitro | Oral administration | 200 mM; 4.4 g/kg | - | - | (140-142) |
|  | Acetate | Mice | DSS-induced Mice model of colitis | In vivo | Oral administration | 4.7% dietary fiber content | - | - | (143) |
|  | VD | Zebrafish | VD-deficient animal models | In vivo | Oral administration | 800 IU/kg | - | - | (144) |
|  | Microalgae | Zebrafish | Soybean meal-induced Zebrafish model | In vivo | Oral administration | 10 g/kg | - | - | (145) |
|  | Altechromone A | Zebrafish | TNBS-induced Zebrafish model of IBD | In vivo | drinking freely | 12.5, 25 and 50 μg/mL | - | - | (146) |
| Natural medicinal materials | Aucklandiae Radix | Mice; cells | DSS-induced Mice model of UC | In vivo and in vitro | Administrated with vehicle | 75 and 300 mg/kg; 0.25, 0.5 and 1 μg/mL | - | LD50 (intragastric administration)-5.87 g/kg; LD50 (intraperitoneal injection)-0.29 g/kg | (147) |
|  | Tiliae Flos | Cells | LPS-stimulated human neutrophils model | In vitro | - | 25, 50, 100, and 200 μg/mL | - | - | (148) |
|  | Phyllanthus niruri Linn | Rats | 1, 2 DMH-induced model of CRC in Rats | In vivo | Oral administration | 13.5 mg/kg | - | - | (149) |
|  | Ethanol extract of cicer arietinum | Mice | DSS-induced Mice model of colitis | In vivo | Oral administration | 100 and 200 mg/kg | - | - | (150) |
|  | Black raspberries | Mice | A mouse model of colorectal cancer (ApcMin/+) | In vivo | Oral administration | 5% | - | - | (151) |
|  | Hydroethanolic extract of fritillariae thunbergii Bulbus | Mice; cells | DSS-induced Mice model of UC | In vivo and in vitro | Oral administration | 100 and 200 mg/kg; 0, 6.25, 12.5 and 25 μg/mL | - | - | (152) |
|  | Ilex rotunda Thunb. | Mice; cell lines | DSS-induced Mice model of UC | In vivo and in vitro | Oral administration | 1.8 g/kg; 120 μg/mL | - | - | (153) |
|  | Hydroalcoholic extract of Araucaria sp. | Rats | TNBS-induced model of colitis in Rats | In vivo | Oral gavage | 30, 100 and 300 mg/kg | - | - | (154) |
|  | Bixa orellana Leaf | Rats | AA-induced model of UC in Rats | In vivo | Oral administration | 100, 200 and 400 mg/kg | - | LD50 (intraperitoneal administration)->1000mg/kg | (155) |
|  | A phytopharmaceutical combining sage and bitter apple | Mice | DSS-induced Mice model of colitis | In vivo | Oral gavage | 1, 10 and 100 mg/kg | - | - | (156) |
| Traditional Chinese medicine compounds | Wu-Mei-Wan | Mice | TNBS-induced Mice model of colitis | In vivo | Oral administration | 0.192 g/kg | - | - | (157) |
|  | Huang Qin Decoction | Mice | AOM/DSS-induced Mice model of colitis-associated cancer | In vivo | Oral administration | 500, 1000 and 1500 mg/kg | - | - | (158) |
|  | Qu-Yu-Jie-Du Decoction | Mice | DSS-induced Mice model of colitis | In vivo | Oral gavage | 4.44 mg/g | - | - | (159) |
|  | Huanglian Ganjiang Decoction | Mice | DSS-induced Mice model of acute colitis | In vivo | Oral administration | CP-2.7 g/kg, AZ-3.2 g/kg, CPAZ-5.9 g/kg | - | - | (160) |
|  | Pulsatilla decoction | Mice | DSS-induced Mice model of colitis | In vivo | Oral gavage | 150 and 300 mg/kg; 20, 40 and 80 mg/kg | - | - | (161, 162) |
|  | Sijunzi Decoction | Mice; human cells | DSS-induced Mice model of UC | In vivo and in vitro | Oral gavage | 400 and 800 mg/kg | - | - | (163) |

**References**

1. Kheir MM, Wang Y, Hua L, Hu J, Li L, Lei F, et al. Acute toxicity of berberine and its correlation with the blood concentration in mice. Food Chem Toxicol. (2010) 48(4):1105-10. doi: 10.1016/j.fct.2010.01.033.
2. Wang M, Liu J, Zhou B, Xu R, Tao L, Ji M, et al. Acute and sub-chronic toxicity studies of Danshen injection in Sprague-Dawley rats. J Ethnopharmacol. (2012) 7;141(1):96-103. doi: 10.1016/j.jep.2012.02.005
3. Dong B, Wu L, Chen Q, Xu W, Li D, Han D, et al. Tolerance Assessment of <i>Atractylodes macrocephala</i> Polysaccharide in the Diet of Largemouth Bass (<i>Micropterus salmoides</i>). Antioxidants (Basel). (2022) 11(8):1581. doi:10.3390/antiox11081581
4. Kuo PC, Hung HY, Nian CW, Hwang TL, Cheng JC, Kuo DH, et al. Chemical Constituents and Anti-inflammatory Principles from the Fruits of Forsythia suspensa. J Nat Prod. (2017) 80(4):1055-1064. doi: 10.1021/acs.jnatprod.6b01141
5. D.Y. Mao, Z.W. Zhang, M. Yang, H.C. Wang, G.J. Wu. Acute toxicity and the induction on IFN-α of forsythiaside in mice. Prog. Vet. Med. (2009) 6:15-17
6. Tan KL, Ali A, Du Y, Fu H, Jin HX, Chin TM, et al. Synthesis and evaluation of bisbenzylidenedioxotetrahydrothiopranones as activators of endoplasmic reticulum (ER) stress signaling pathways and apoptotic cell death in acute promyelocytic leukemic cells. J Med Chem. (2014) 57(14):5904-18. doi: 10.1021/jm401352a
7. Krishnaraju AV, Sundararaju D, Sengupta K, Venkateswarlu S, Trimurtulu G. Safety and toxicological evaluation of demethylatedcurcuminoids; a novel standardized curcumin product. Toxicol Mech Methods. (2009) 19(6-7):447-460. doi:10.1080/15376510903200766
8. Park EJ, Kim Y, Kim J. Acylated flavonol glycosides from the flower of Inula britannica. J Nat Prod. (2000) 63(1):34-6. doi: 10.1021/np990271r
9. Parlar A, Arslan SO, Çam SA. Glabridin Alleviates Inflammation and Nociception in Rodents by Activating BK<sub>Ca</sub> Channels and Reducing NO Levels. Biol Pharm Bull. (2020) 43(5):884-897. doi:10.1248/bpb.b20-00038
10. Liu YW, Cheng YB, Liaw CC, Chen CH, Guh JH, Hwang TL, et al. Bioactive diterpenes from Callicarpa longissima. J Nat Prod. (2012) 75(4):689-93. doi: 10.1021/np200932k
11. Mishra, V., Soren, A.D., Yadav, A.K. Toxicological evaluations of betulinic acid and ursolic acid; common constituents of Houttuynia cordata used as an anthelmintic by the Naga tribes in North-east India. Futur J Pharm Sci. (2021) 7: 39. [doi:10.1186/s43094-020-00173-4](https://doi.org/10.1186/s43094-020-00173-4)
12. Wang XH, Zhou SY, Qian ZZ, Zhang HL, Qiu LH, Song Z, et al. Evaluation of toxicity and single-dose pharmacokinetics of intravenous ursolic acid liposomes in healthy adult volunteers and patients with advanced solid tumors. Expert Opin Drug Metab Toxicol. (201) 9(2):117-125. doi:10.1517/17425255.2013.738667
13. Cosmetic Ingredient Review Expert Panel. Final report on the safety assessment of Glycyrrhetinic Acid, Potassium Glycyrrhetinate, Disodium Succinoyl Glycyrrhetinate, Glyceryl Glycyrrhetinate, Glycyrrhetinyl Stearate, Stearyl Glycyrrhetinate, Glycyrrhizic Acid, Ammonium Glycyrrhizate, Dipotassium Glycyrrhizate, Disodium Glycyrrhizate, Trisodium Glycyrrhizate, Methyl Glycyrrhizate, and Potassium Glycyrrhizinate. Int J Toxicol. (2007) 26 Suppl 2:79-112. doi: 10.1080/10915810701351228
14. Q. Gong, M. Wang, L. He, Y. Feng, S. Yang, L. Du, et al. Protective effect of anemoside B4 from experimental acute renal damage in animal. Chin. Mod. Med. (2019) 21 (01):62-67+70. doi: 10.13313/j.issn.1673-4890.20180703001
